# In Vitro Anti-Breast Cancer Effects of *Tamarix aphylla*-Derived Quercetin and In Silico Insights into Its Targeting of PIP4K2A

**DOI:** 10.3390/ijms27157063

**Published:** 2026-08-06

**Authors:** Dhurgham Al-Fahad, Zahraa Naeem Hashim, Suliman A. Almahmoud, Faizul Azam

**Affiliations:** 1 Department of Pathological Analysis, College of Science, University of Thi-Qar, Nasiriyah 64001, Iraq; dhurgham.alfahad@utq.edu.iq (D.A.-F.); zahraa.naeem@utq.edu.iq (Z.N.H.); 2 Department of Pharmaceutical Chemistry and Pharmacognosy, College of Pharmacy, Qassim University, Buraydah 51452, Saudi Arabia; s.almahmoud@qu.edu.sa

**Keywords:** in silico, quercetin, PIP4K2A, in vitro

## Abstract

Phosphatidylinositol 5-phosphate 4-kinase type 2 alpha (PIP4K2A) is a key oncogenic driver that regulates the PI5P/PIP2 axis to promote metastatic migration in breast cancer. This study aimed to investigate the therapeutic potential of a crude extract from *Tamarix aphylla* against breast cancer progression and identify its primary active constituents. The crude extract was initially evaluated against MDA-MB-231 and MCF7 breast cancer cell lines using wound healing assays. Bioassay-guided isolation and screening were deployed to isolate individual components, and the most potent lead compound was structurally characterized using preparative HPLC and FTIR. To analyze its interaction with PIP4K2A, in silico molecular docking, MM/GBSA calculations, and 200 ns molecular dynamics simulations were conducted. In vitro validation was subsequently performed via dose-dependent cytotoxicity assays, scratch assays, single-cell tracking, and RT-qPCR expression analysis. Quercetin was identified as the most potent lead inhibitor against PIP4K2A. Computational modeling revealed that quercetin binds tightly within the PIP4K2A ATP-binding pocket, yielding a superior binding affinity of −10.77 kcal/mol and enhanced thermodynamic stability (ΔGMM/GBSA = −42.6 ± 2.1 kcal/mol) compared to the native ligand (ΔG MM/GBSA = −23.3 ± 1.8 kcal/mol). Molecular dynamics simulations confirmed an induced-fit structural transition that locked the complex into an ultra-stable conformation within a deep global energy minimum basin (−10.8 kcal/mol). In vitro assays demonstrated dose-dependent cytotoxicity, with aggressive triple-negative MDA-MB-231 cells exhibiting higher sensitivity (IC_50_ = 82.23 µg/mL) than luminal MCF7 cells (IC_50_ = 97.14 µg/mL). Furthermore, scratch and single-cell tracking assays showed a profound suppression of migration speed and wound closure (reduced to ~40%), while RT-qPCR revealed a near-complete transcriptional knockdown of PIP4K2A mRNA expression (down to 0.025-fold). Collectively, these findings elucidate a unique dual-action mechanism for *Tamarix aphylla*-derived quercetin—characterized by both direct competitive enzymatic inhibition and downstream transcriptional silencing—positioning it as a promising therapeutic scaffold for targeted anti-metastatic breast cancer interventions.

## 1. Introduction

Breast cancer persists as a leading cause of cancer-related morbidity and mortality worldwide, presenting a formidable challenge to contemporary oncology due to its high heterogeneity and metastatic propensity [1,2]. Emerging evidence highlights the deregulation of protein and lipid kinases as central drivers of the aberrant signaling networks that orchestrate tumor cell survival, proliferation, and metabolic reprogramming [3,4]. Consequently, identifying novel, hyper-targeted kinase inhibitors remains a primary focus for overcoming the limitations and systemic toxicities associated with conventional chemotherapies [5,6].

Within the landscape of oncogenic lipid signaling, Phosphatidylinositol 5-phosphate 4-kinase type 2 alpha (PIP4K2A) has emerged as a crucial metabolic and metastatic hub [7,8]. PIP4K2A catalyzes the ATP-dependent phosphorylation of phosphatidylinositol 5-phosphate (PI5P) to generate phosphatidylinositol 4,5-bisphosphate (PIP2) [9,10]. This enzymatic step controls the intracellular pools of these critical phosphoinositide messengers [11,12]. Elevated PIP4K2A activity and frequent somatic mutations are heavily correlated with aggressive tumor phenotypes, where the enzyme modulates focal adhesion dynamics to accelerate cellular locomotion and directional invasion [13]. Conversely, the accumulation of its substrate, PI5P, resulting from PIP4K2A downregulation or targeted inhibition, triggers cellular stress, suppresses growth factor signaling, and impairs the cytoskeletal remodeling required for metastasis [14,15]. Thus, pharmacological targeting of PIP4K2A offers an innovative strategy to disrupt the metastatic cascade at its molecular origin [16].

Concurrently, natural products continue to serve as an indispensable repository for novel small-molecule scaffolds due to their structural diversity and multi-targeted efficacy [17]. *Tamarix aphylla*, a halophytic plant native to arid regions, is rich in bioactive polyphenols and flavonoids. Among these, quercetin (a ubiquitous pentahydroxyflavone) has garnered considerable attention for its broad-spectrum anticancer, anti-inflammatory, and antioxidant properties [18]. Although prior literature suggests that quercetin can modulate broad intracellular networks such as the PI3K/Akt axis [19], its precise structural interaction and inhibitory potential against non-classical lipid kinases like PIP4K2A remain completely unexplored.

**Figure 1:**
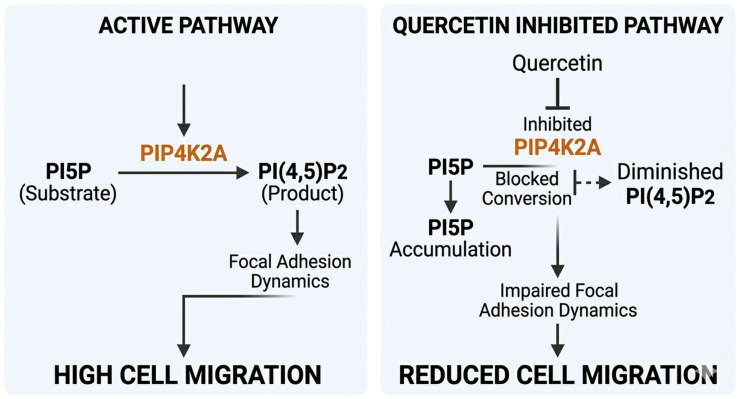
Proposed molecular mechanism of Quercetin-mediated inhibition of breast cancer cell migration. Solid arrows (→) denote pathway progression, enzymatic reaction, or activation; blunt-ended lines (⊥) denote inhibition; and dashed arrows (⇢) indicate reduced or impaired molecular conversion flux.

This study targets the oncogenic enzyme PIP4K2A, which typically converts phosphatidylinositol 5-phosphate (PI5P) into phosphatidylinositol 4,5-bisphosphate (PI(4,5)P_2_) to stimulate focal adhesion dynamics and accelerate metastatic breast cancer cell migration. By introducing quercetin as a targeted inhibitor, the catalytic activity of PIP4K2A is blocked, resulting in a diminished pool of downstream PI(4,5)P_2_ and an upstream accumulation of unbiotransformed PI5P substrate. This targeted disruption ultimately impairs downstream focal adhesion dynamics, culminating in the profound suppression of cell migration velocity and wound-healing kinetics (Figure 1).

To optimize this discovery pipeline, advanced in silico computational workflows—including molecular docking, molecular dynamics (MD) simulations, and Molecular Mechanics/Generalized Born Surface Area (MM/GBSA) energetics—have become vital tools to rapidly decode ligand-receptor mechanics prior to wet-lab execution [20,21,22].

In this study, we present an integrated, cross-disciplinary strategy that positions *Tamarix aphylla*-derived quercetin as a highly potent inhibitor of PIP4K2A. By coupling rigorous computational physics with phenotypic and molecular in vitro assays, we demonstrate that quercetin effectively suppresses breast cancer migration and proliferation via a distinct dual-action mechanism involving direct competitive active-site binding and potent transcriptional gene silencing.

## 2. Results

### 2.1. Analytical Isolate Profiling from Tamarix aphylla Extracts

Chromatographic resolution of the *Tamarix aphylla* ethyl acetate extract via high-performance liquid chromatography (HPLC) revealed a clean, well-resolved multi-component profile. Peak comparisons with highly pure commercial reference standard standards identified four major flavonoid fractions: (~3.94 min; Supplementary Figure S2), Rutin (~4.88 min; Supplementary Figure S3), Quercetin (~8.21 min; Supplementary Figure S4), and Kaempferol (~9.97 min; Supplementary Figure S5). FTIR spectral characterization further confirmed the structural identity of the isolates, matching standard vibration frequencies. Although all four compounds exhibited notable properties, quercetin was selected as the primary lead scaffold for structural validation due to its superior score during initial target-docking screens against the lipid kinase active site.

### 2.2. Molecular Docking In Silico and Intermolecular Interaction Mapping

To optimize the experimental workflow and focus resources on the most promising candidates, all four isolated compounds were initially screened against the ATP-binding cavity of the core oncogenic target, PIP4K2A, and evaluated against the native co-crystallized ligand. Quercetin emerged as the primary lead scaffold, demonstrating superior binding affinity and computational metrics compared to the other isolated flavonoids. Specifically, quercetin exhibited a high binding potential characterized by a docking score of −10.0 kcal/mol and an exceptional relative binding free energy ${\Delta G}^{bind}$ −42.6 calculated via MM/GBSA. Structural analysis revealed that this affinity is driven by a network of short, conventional hydrogen bonds with key hinge-region residues, VAL199 and ASN198, within the catalytic pocket. This robust interaction profile positions quercetin as a potent candidate capable of achieving steady-state competitive enzymatic inhibition and downstream structural stabilization.

**Figure 2:**
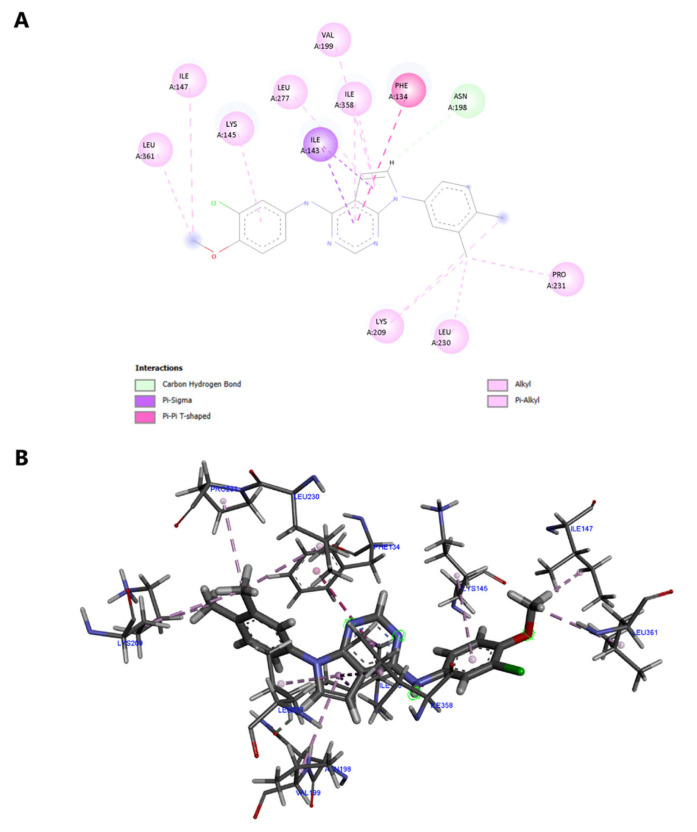
Molecular interaction profiling of the co-crystal ligand–PIP4K2A complex. (**A**): the 2D interaction diagram detailing key non-covalent contacts, including a π–sigma interaction with ILE143 (purple), a π–π T-shaped interaction with PHE134 (dark pink), a carbon–hydrogen bond with ASN198 (light green), and alkyl π–alkyl hydrophobic interactions with LYS145, ILE147, VAL199, LYS209, LEU230, PRO231, LEU277, ILE358, and LEU361 (light pink). (**B**): the corresponding 3D binding conformation, highlighting the spatial arrangement and geometric orientation of the ligand relative to the active-site amino acid residues.

**Figure 3:**
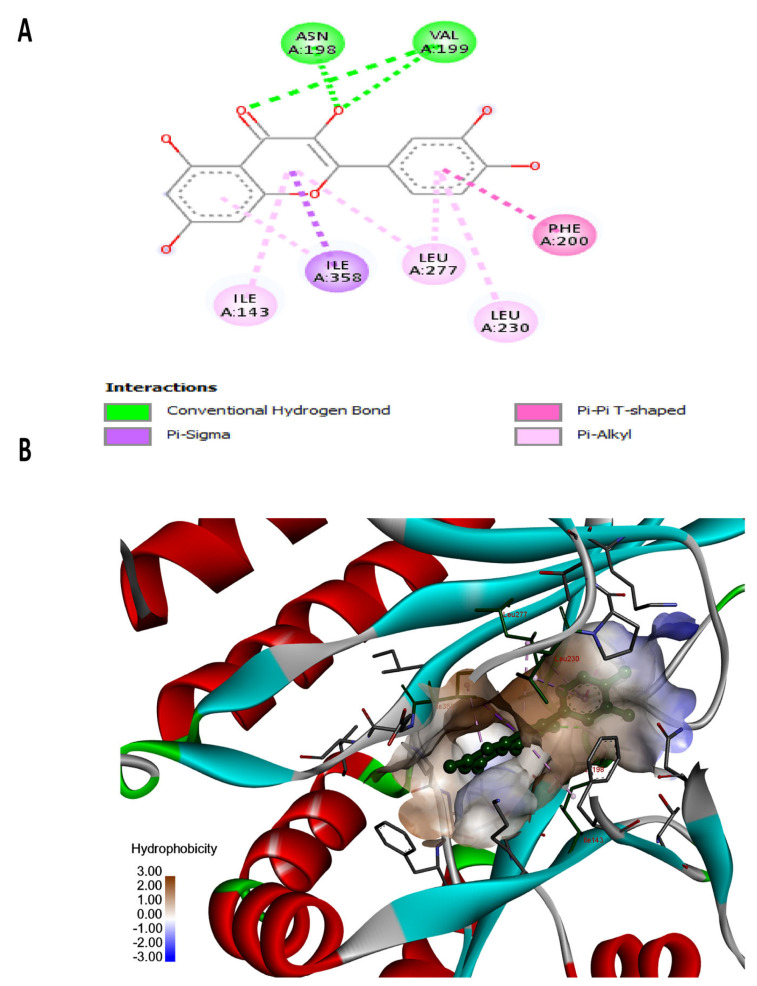
Molecular interaction profiling of Quercetin within the PIP4K2A active site. (**A**): 2D schematic displaying the non-covalent interaction network, including hydrogen bonds, π–sigma, and π-alkyl interactions. Interaction frequency map highlighting high-occupancy hydrogen bonds with Val199 (91%) and Asn198 (87%), along with a water-bridged interaction to Thr196 (30%). (**B**): 3D binding mode showing Quercetin (green sticks) surrounded by key catalytic pocket residues (gray lines).

The native ligand lacks classical hydrogen bonding, relying instead on weaker carbon–hydrogen interactions with ASN 198 (99% distance) and a water-bridged network with THR 196 (35%) (Figure 2B). In contrast, quercetin forms strong, conventional hydrogen bonds with the backbone of key hinge-region residues VAL 199 (91% occupancy distance) and ASN 198 (87% occupancy distance) (Figure 3A).

Furthermore, quercetin utilizes its rigid polyphenolic core to optimize steric complementarity within the binding pocket, forming clean hydrophobic and aromatic networks (Figure 3B). These include a strong π–π T-shaped alignment with PHE 200 and a tightly integrated water-mediated bridge linking THR 196 (30%) and VAL 199 (36%).

Conversely, the native ligand’s aromatic contacts with PHE 134 (38%) and its π -sigma interaction with VAL 199 (86%) operate at less favorable spatial distances (Figure 2). This structural shift from weak, unstable carbon–hydrogen contacts to short, conventional hydrogen bonds positions quercetin as a highly promising therapeutic candidate. Re-docking of the co-crystallized ligand into PIP4K2A faithfully reproduced its binding interactions, validating the computational protocol. Virtual screening identified Quercetin as the primary lead scaffold, demonstrating superior binding affinity (−10.77 kcal/mol ΔG = −42.6 kcal/mol) (Supplementary Figure S6), compared to co-crystallized ligand (−9.02 kcal/mol). Quercetin’s high affinity was driven by key conventional hydrogen bonds with hinge residues ASN198 (87% occupancy) and VAL199 (91% occupancy), stabilized by π–π stacking with PHE200 and hydrophobic contacts (ILE143, LEU230, LEU277, and ILE358) (Supplementary Figure S7).

In silico mutagenesis of hinge residues ASN198 and VAL199 to alanine validated their critical role in stabilizing Quercetin, causing its binding affinity to drop significantly from −10.77 kcal/mol (RMSD: 1.41 Å) in the wild-type to −8.57 kcal/mol (RMSD: 0.61 Å) in the N198A/V199A mutant (Table 1). Structural analysis revealed that this 2.20 kcal/mol binding penalty was primarily driven by the complete loss of key backbone hydrogen bonds at position 198 and structural rearrangements introducing unfavorable donor-donor repulsions with LYS209 (Supplementary Figure S8), even though core hydrophobic contacts with residues such as PHE134, PHE200, and LEU230 were preserved. Conversely, the reference co-crystallized ligand retained high binding affinity in the mutant model at −9.90 kcal/mol (RMSD: 1.31 Å) compared to −9.02 kcal/mol (RMSD: 1.17 Å) in the wild-type (Table 1), forming alternative hydrogen bonds with ALA199 and THR196 alongside key hydrophobic contacts (Supplementary Figure S9). Because co-crystallized ligand maintained strong binding within the mutated pocket, these findings demonstrate that the reduced affinity of Quercetin is ligand-specific rather than a consequence of global cavity disruption, conclusively establishing ASN198 and VAL199 as indispensable hotspot anchors required for high-affinity Quercetin binding.

### 2.3. Molecular Dynamics (MD) Tracking and Structural Rigidity Calculations

**Figure 4:**
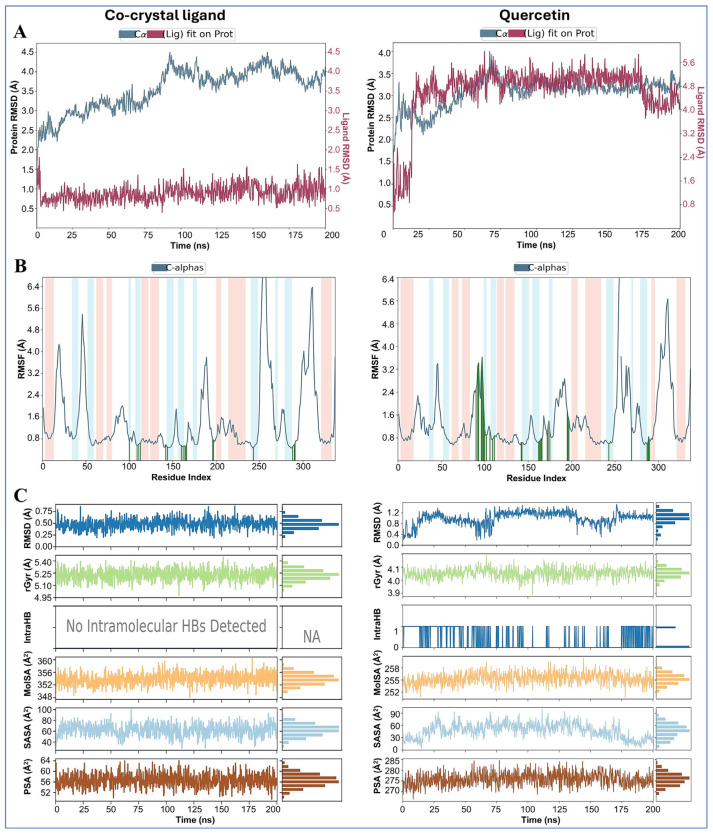
Comparative molecular dynamics (MD) simulation profiles of the target protein bound to the co-crystallized ligand (left) and quercetin (right) over a 200 ns trajectory. (**A**) Protein backbone and ligand Root Mean Square Deviation (RMSD) plots showing stable protein backbone dynamics (~3.0–3.5 Å) across both complexes, confirming overall tertiary fold retention. Quercetin undergoes an initial induced-fit structural reorientation (RMSD ~3.2–4.5 Å) during the first 15–25 ns—characterized by B-ring rotation relative to the chromone core to optimize hydrogen bonding with hinge residues ASN198 and VAL199—before stabilizing into a plateau. (**B**) Root Mean Square Fluctuation (RMSF) profiles annotated with secondary structure elements (pink bars: α-helices; blue bars: β-strands; green vertical lines: key ligand-contact residues). Quercetin induces controlled, localized flexibility in adjacent loop regions (residues 90–100, RMSF peak ~3.6 Å) to facilitate binding pocket lock-in within a deeper energetic basin. (**C**) Time-dependent monitoring of structural integrity metrics—including radius of gyration (Rg, maintained at 20.9–21.1 Å, indicating high compactness), intramolecular hydrogen bonds, solvent-accessible surface area (SASA), and polar surface area (PSA)—demonstrating that quercetin remains deeply sequestered and shielded within the hydrophobic catalytic cleft throughout the 200 ns simulation.

To evaluate the long-term stability and structural endurance of the protein–ligand complexes under simulated physiological conditions, the docking configurations were subjected to 200 ns unrestrained molecular dynamics (MD) simulations. Root-mean-square deviation (RMSD) analysis revealed distinct dynamic behaviors for each system (Figure 4A). Both protein backbones maintained a low, steady RMSD (~3.0–3.5 Å), demonstrating that ligand binding preserves overall tertiary fold stability without causing global denaturation or unnatural structural distortion. However, ligand RMSD trajectories diverged significantly: while the native ligand remained locked in a rigid, static pose (average RMSD ~0.9 Å), the Quercetin-bound complex underwent an early structural adjustment between 15 and 25 ns, stabilizing into a plateau at approximately 4.5 Å (Figure 4A).

This local adaptation is further highlighted by the root-mean-square fluctuation (RMSF) profiles (Figure 4B). While the structural backbone elements remained rigid, Quercetin induced controlled, localized flexibility in adjacent loop regions (residues 90–100, peaking at ~3.6 Å), facilitating the active-site rearrangement necessary to lock the molecule into a deeper thermodynamic binding basin. Analysis of secondary structure elements in Figure 4B (represented by pink shaded regions for alpha-helices and blue shaded regions for beta-strands/loops) confirms that elevated fluctuations were strictly confined to flexible loop segments rather than core secondary frameworks, while green vertical bars illustrate an expanded network of stabilizing ligand contacts across the pocket. Finally, time-dependent structural integrity metrics (Figure 4C) confirmed the overall compactness and shielding of the complex; stable Radius of Gyration values (Rg approx 20.9_ 21.1 Å) combined with consistently low Solvent Accessible Surface Area (SASA) and Polar Surface Area (PSA) demonstrate that Quercetin remains tightly bound, deeply sequestered, and shielded within the hydrophobic catalytic cleft throughout the entire 200 ns trajectory.

**Figure 5:**
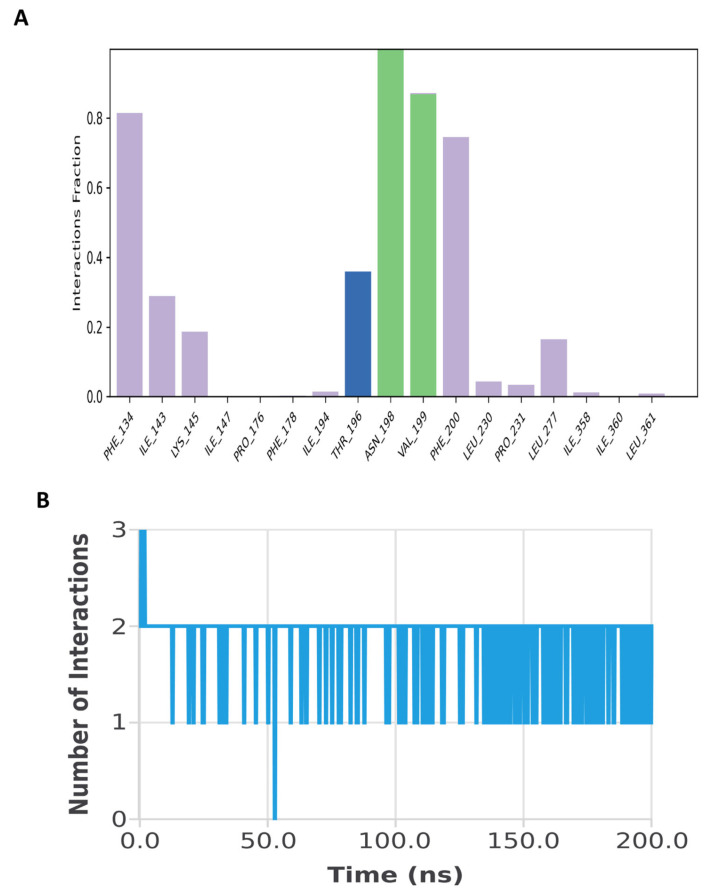
Interaction stability and contact frequency of the PIP4K2A-Co-Crystal Ligand complex. (**A**) Protein–Ligand Contacts: Normalized interaction fractions per residue over the simulation course. Bars are color-coded by type: hydrophobic (purple), water bridges (blue), and hydrogen bonds (green), with values above 1.0 indicating multiple simultaneous interactions. Main stabilizing contributors include PHE_134, ASN_198, VAL_199, and PHE_200. (**B**) Interaction Timeline: Total number of specific protein–ligand interactions maintained over the 200 ns trajectory, demonstrating a stable binding profile that consistently maintains 1 to 2 concurrent contacts.

**Figure 6:**
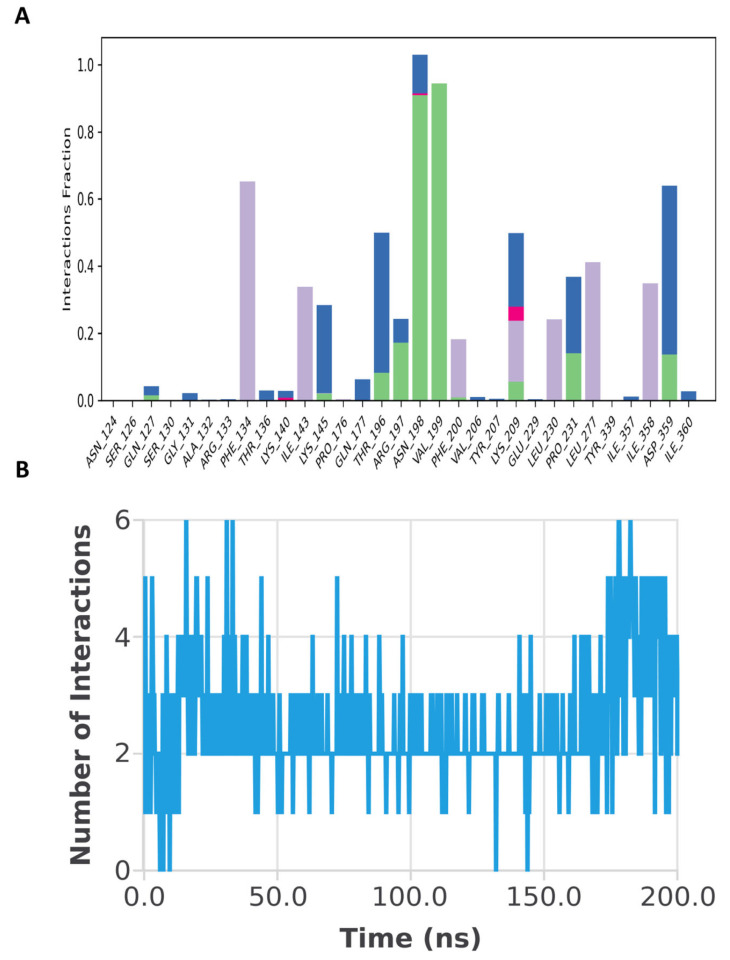
Interaction stability and contact frequency of the PIP4K2A- Quercetin complex. (**A**) Protein–Ligand Contacts: Normalized interaction fractions per residue over the simulation course. Bars are color-coded by interaction type: hydrophobic (purple), water bridges (blue), hydrogen bonds (green), and ionic interactions (pink). Values above 1.0 (e.g., ASN_198) indicate multiple simultaneous interactions. Key stabilizing residues include PHE_134, THR_196, ASN_198, VAL_199, LYS_209, and ASP_359. (**B**) Interaction Timeline: Total number of specific protein–ligand interactions tracked over the 200 ns trajectory, showing a robust binding profile that dynamically fluctuates primarily between 2 and 5 concurrent contacts.

Analysis of contact frequencies over time revealed that the co-crystallized refer-ence ligand was heavily restricted to a maximum of 2 to 3 simultaneous contacts (Fig-ure 5B), driven primarily by fixed interactions with PHE 134, ASN 198, and VAL 199 (Figure 5A). In contrast, quercetin sustained a highly dynamic interaction network, consistently overcoming this rigid dual-contact limitation (Figure 6). Throughout the course of the trajectory, quercetin maintained a fluid profile of 2 to 6 simultaneous hy-drogen bonds (Figure 6B), with ASN 198 and VAL 199 acting as primary stabilizing anchor residues. This versatile, multi-point engagement allowed its peripheral hy-droxyl groups to constantly recruit additional nearby residues within the binding pocket, including ARG 197, LYS 209, PRO 231, and ASP 359 (Figure 6A). Furthermore, quercetin established a robust, prolonged water-mediated bridge profile at positions THR 196 and ASP 359, providing a clear structural explanation for its enhanced ther-modynamic binding affinity.

**Figure 7:**
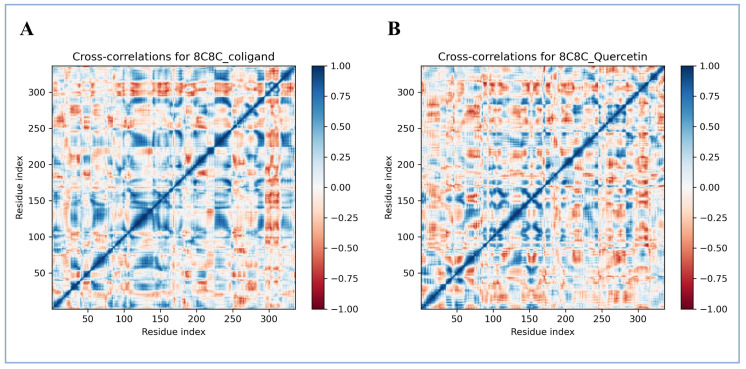
Dynamic cross-correlation matrices (DCCM) of the PIP4K2A protein complexes. The heatmaps depict the collective fluctuations and internal dynamics of residue pairs for PIP4K2A _coligand (**A**) and PIP4K2A _Quercetin (**B**) over the simulation trajectory. The axes correspond to residue indices (1–330). The color scale ranges from −1.00 to 1.00, where blue indicates highly correlated motions (residues moving in the same direction), red indicates strongly anti-correlated motions (residues moving in opposite directions), and white represents completely uncorrelated motions. Differences in the off-diagonal blocks highlight how each distinct ligand modulates the global conformational flexibility and domain movements of the protein.

To evaluate large-scale domain movements, Dynamic Cross-Correlation Matrices (DCCM) were constructed. Quercetin induced strong anti-correlated motions (indicated by intense cyan/blue zones), demonstrating that it tightens the enzyme’s global architecture more efficiently than the native ligand. This compact state was confirmed by the Radius of Gyration (R_g), which remained remarkably steady between 20.9 and 21.1Å throughout the simulation (Figure 7).

**Figure 8:**
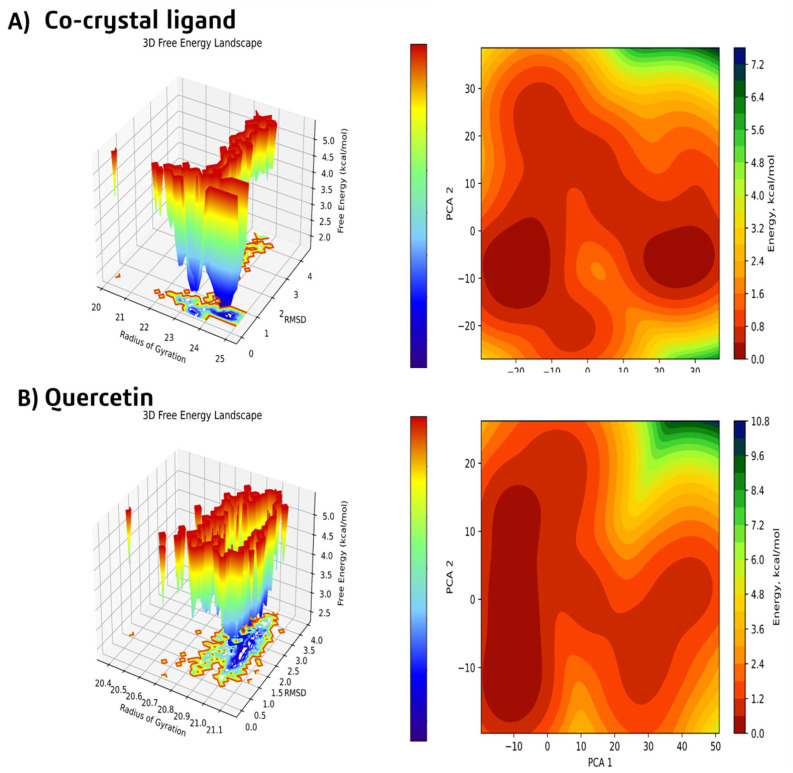
Free energy landscapes (FEL) and principal component analysis (PCA) of the protein complexes. Profiles are shown for the crystal ligand (**A**) and Quercetin (**B**) complexes. Left Panels (3D FEL): Thermodynamic landscapes mapped as a function of Radius of Gyration and RMSD. The deep blue basins define the global energy minima, representing the most stable, favored conformational states. Right Panels (2D PCA): Free energy contour maps projected along the first two principal components (PCA 1 and PCA 2). The color scale (kcal/mol) indicates relative free energy, where deep red basins correspond to the lowest energy states and most highly populated structural ensembles.

Furthermore, Principal Component Analysis (PCA) and Free Energy Landscape (FEL) mapping confirmed that the Quercetin-bound complex achieves superior thermodynamic convergence compared to the co-crystallized native ligand (Figure 8). Quercetin settled into a single, unified, and narrow global energy minimum basin with a depth of ΔG = −10.8 kcal/mol (Figure 8B). In accordance with energy landscape theory, this single deep energy funnel demonstrates that the ligand–protein complex achieves clean thermodynamic convergence, effectively trapping and locking the enzyme into a single, highly stable conformation (Figure 8B). In contrast, the native ligand’s landscape was characterized by fragmented, multi-peaked energy states averaging only −7.2 kcal/mol (Figure 8A), featuring two discrete, separated global minima. This structural bifurcation signifies conformational meta-stability, where the co-crystal ligand continuously hops between two distinct sub-states within the catalytic pocket during simulation (Figure 8A). Ultimately, this significantly deeper energy basin confirms that the initial induced-fit transition locks Quercetin into a thermodynamically favored, highly stable, and strongly inhibited state (Figure 8).

### 2.4. In Vitro Cytotoxicity and Selectivity Determinants

**Figure 9:**
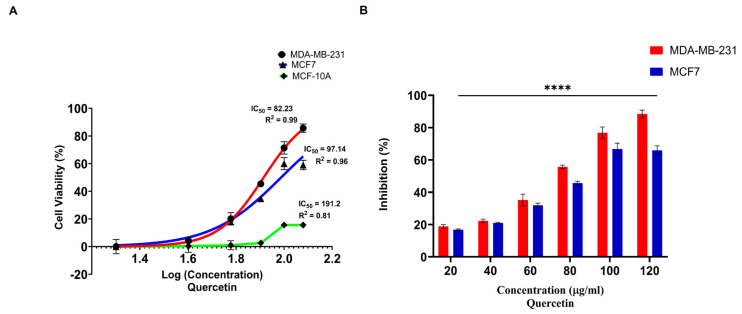
In vitro cytotoxic effects and IC_50_ profiles of quercetin against breast cancer and normal cell lines. (**A**) Dose–response curves: Non-linear regression analysis plotting cell response against the log concentration of quercetin to determine half-maximal inhibitory concentration (IC_50_) values. The iso-lated compound exhibited high selectivity toward cancer cells, yielding IC_50_ values of 82.23 μg/mL (R^2^ = 0.99) for MDA-MB-231 (red line) and 97.14 μg/mL (R^2^ = 0.96) for MCF7 (blue line), while demonstrating significantly lower toxicity against the normal human breast epithelial cell line MCF-10A (IC_50_ = 191.2 μg/mL, R^2^ = 0.81). (**B**) Comparative growth inhibition: Bar graph quantifying the percentage of growth inhibition in MDA-MB-231 (red bars) and MCF7 (blue bars) cells across a linear concentration range (20–120 μg/mL). Quercetin induces a clear dose-dependent increase in cellular inhibition in both malignancies. Data are presented as mean ± SD from three independent experiments (*n* = 3). Highly significant differences across treatment groups are indicated by asterisks (**** *p* < 0.0001).

Colorimetric MTT viability assays demonstrated that quercetin exerts robust, dose-dependent cytotoxicity against both human breast cancer cell models. Notably, the highly metastatic, triple-negative breast cancer (TNBC) cell line MDA-MB-231 displayed a higher sensitivity to quercetin (IC_50_ = 82.23 μg/mL) compared to the estrogen receptor-positive MCF7 cell line (IC_50_ = 97.14 μg/mL) (Figure 9). Importantly, quercetin exhibited significantly lower toxicity against the non-tumorigenic human breast epithelial cell line MCF-10A, yielding a substantially higher (IC_50_ = 191.2 μg/mL R^2^ = 0.81), thereby indicating a favorable selectivity profile toward malignant cells (Figure 9). This enhanced susceptibility in TNBC cells highlights quercetin’s potential to target aggressive phenotypes that lack standard hormonal receptors, making it a promising candidate for targeted oncology strategies [22,23,24].

### 2.5. Attenuation of Collective Wound Healing and Single-Cell Kinematic Velocity

**Figure 10:**
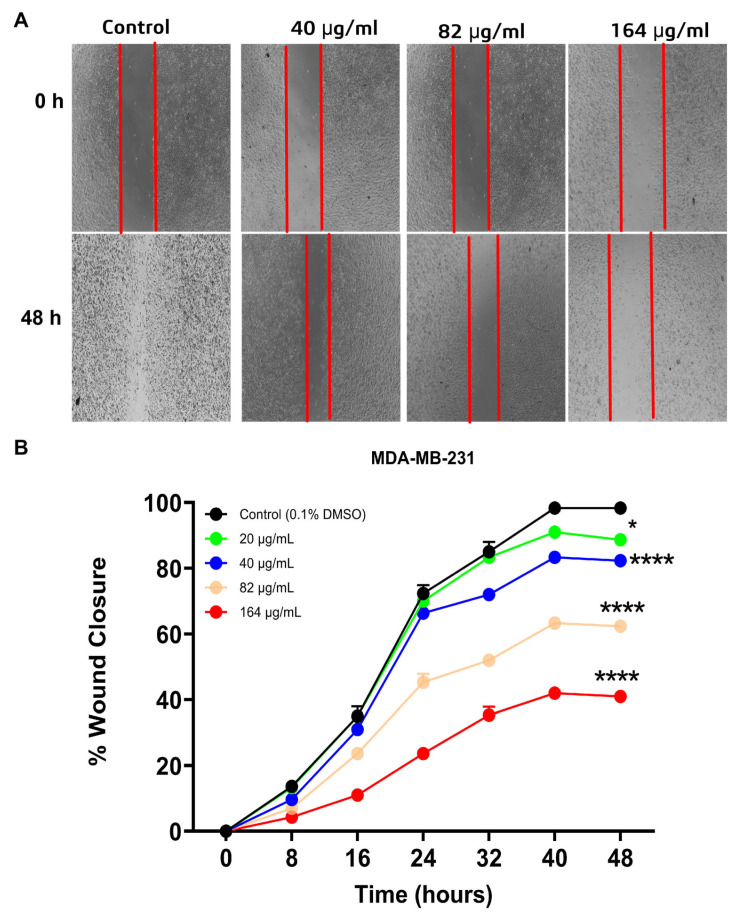
Inhibitory effect of Quercetin on the migratory capacity of MDA-MB-231 breast cancer cells. (**A**). Representative phase-contrast microscopic images of the scratch wound healing assay in MDA-MB-231 cells at baseline (0 h, top row) and after 48 h of treatment (bottom row). Cells were treated with a vehicle control (0.1% DMSO) or increasing concentrations of the compound (20, 40, 82, and 164 μg/mL). The red lines delineate the advancing boundaries of the scratch wound over time. (**B**). Quantitative line graph representing the percentage of wound closure measured at 8 h intervals over a 48 h period. Data points represent the mean ± SD (or SEM) of three independent experiments (*n* = 3). Statistical significance compared to the vehicle control group is indicated by asterisks: * *p* < 0.05 and **** *p* < 0.0001. Scale bar = 100 µm.

**Figure 11:**
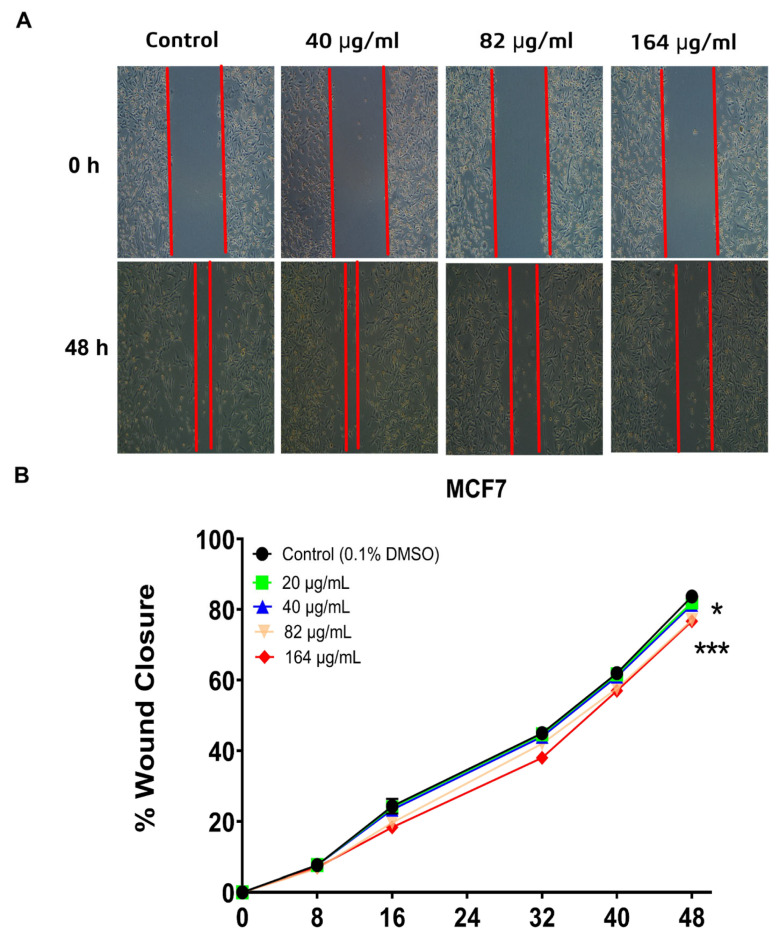
Effect of Quercetin on the migratory capacity of MCF7 breast cancer cells. (**A**). Representative phase-contrast micrographs displaying the scratch wound healing assay in MCF7 cells at baseline (0 h, top row) and after 48 h of incubation (bottom row). Cells were exposed to either the vehicle control (0.1% DMSO) or selected concentrations of the treatment. The red lines mark the migrating borders of the cell monolayer. (**B**). Quantitative representation of the percentage of wound closure tracked over a 48 h period at 8 h intervals. The migration rate of MCF7 cells shows a concentration-dependent deceleration, particularly prominent at the 48 h mark. Data points are expressed as the mean ± SD (or SEM) from three independent replicates (*n* = 3). Statistical significance relative to the vehicle control group is indicated by asterisks: * *p* < 0.05 and *** *p* < 0.001. Scale bar = 100 µm.

To validate the anti-metastatic potential of quercetin suggested by the computational models, in vitro scratch wound healing assays were conducted over a 48 h window. While untreated control cohorts achieved near-complete wound closure, treatment with quercetin resulted in a potent, dose-dependent arrest of cell migration. In the aggressive MDA-MB-231 line, exposure to 164 g/mLof quercetin restricted absolute wound closure to just 40% (*p* < 0.0001), demonstrating strong anti-migratory activity (Figure 10). Interestingly, the luminal MCF7 line exhibited noticeable resistance, showing only minor, late-stage migratory delays at the 48 h mark (*p* < 0.05). This distinction emphasizes the high selectivity of quercetin against highly motile, metastatic cell models (Figure 11).

**Figure 12:**
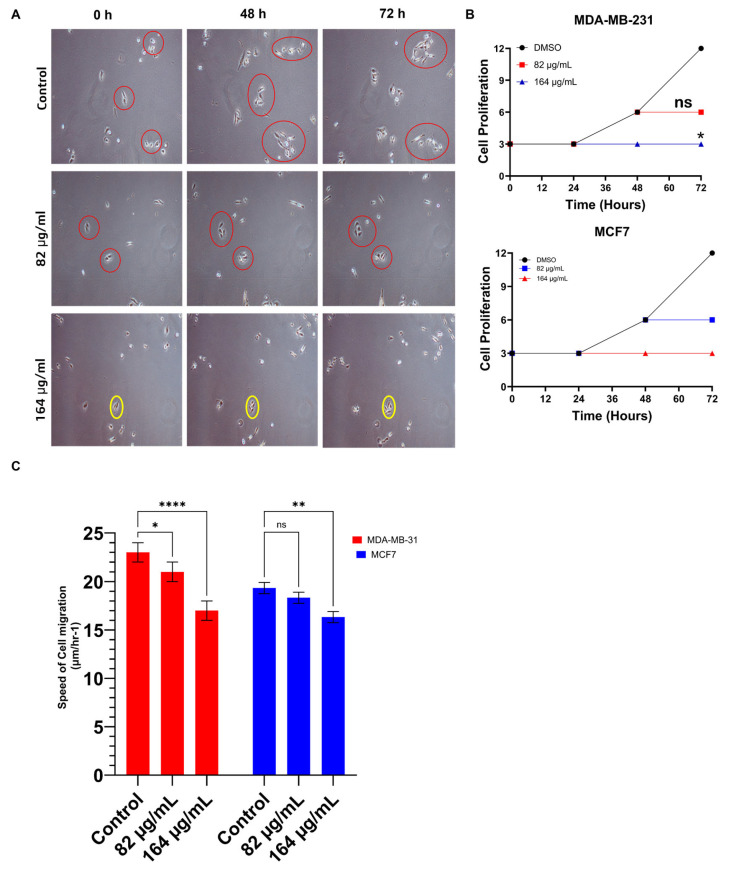
Quercetin suppresses cell proliferation and kinetic migration speed in breast cancer cell lines. (**A**) Representative phase-contrast tracking micrographs illustrating single-cell proliferation dynamics under treated conditions. (**B**,**C**) Proliferation line graphs demonstrating that quercetin significantly arrests cell division over 72 h at 164 μg/mL in both MDA-MB-231 (**B**) and MCF7 (**C**) cells (* *p* < 0.05) compared to exponentially growing DMSO controls. (**D**) Migration speed analysis revealing a potent, dose-dependent reduction in the velocity of metastatic MDA-MB-231 cells (* *p* < 0.05 and **** *p* < 0.0001), whereas the less aggressive MCF7 line experiences a significant reduction in speed only at the highest concentration of 164 μg/mL (*p* < 0.01). Together, these data demonstrate that quercetin exerts robust, statistically significant inhibitory effects on both the growth rate and physical migration speed of breast cancer cells. Data are presented as mean ± SD (N = 50 cells analyzed per group). Statistical significance is indicated by asterisks (* *p* < 0.05, ** *p* < 0.01, **** *p* < 0.0001 vs. control).

To determine whether this phenotypic change resulted from individual locomotor impairment or collective growth arrest, high-resolution single-cell live tracking assays were conducted over 72 h. The tracking data revealed that quercetin simultaneously targets both cellular proliferation and kinetic velocity. At a concentration of 82 g/mL, it exerted a cytostatic effect that completely halted cell division after 48 h. At the highest dose (164 g/mL), it induced profound growth arrest across both cell lines (Figure 12).

Concurrently, cell tracking analysis showed a sharp reduction in individual migration velocity. This impairment of cell velocity was particularly pronounced in the highly motile MDA-MB-231 line. This suggests that quercetin interferes with the key biochemical signaling pathways that govern cytoskeletal reorganization, lamellipodia formation, and epithelial–mesenchymal transition (EMT) factors.

### 2.6. Transcriptional Knockdown of the PIP4K2A Oncogene

**Figure 13:**
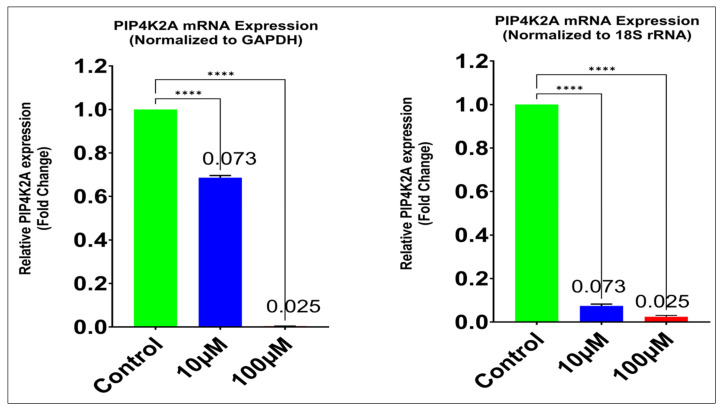
Effect of quercetin on PIP4K2A mRNA expression. **Left**: Relative PIP4K2A expression normalized to GAPDH, showing a stark reduction at 10 µM (0.073-fold) and 100 µM (0.025-fold) compared to the untreated control. **Right**: Relative PIP4K2A expression normalized to 18S rRNA, confirming the drastic, dose-dependent downregulation at identical concentrations. Data are presented as mean ± SD. Statistical significance is indicated by asterisks (**** *p* < 0.0001 vs. control).

To establish a direct link between the observed phenotypic alterations and the proposed molecular target, quantitative real-time PCR (RT-qPCR) was performed to evaluate PIP4K2A mRNA expression levels in MDA-MB-231 cells following treatment. The RT-qPCR analysis revealed a profound, dose-dependent down-regulation of *PIP4K2A* transcripts. When normalized against the *GAPDH* housekeeping gene, exposure to 10 µM of quercetin reduced relative *PIP4K2A* expression to just 0.073-fold compared to untreated controls. Increasing the dose to 100 mu\M caused expression levels to plummet to 0.025-fold. This transcriptional suppression was fully cross-validated when normalized against the *18S rRNA* internal control, confirming the high accuracy of the assay (Figure 13).

By downregulating this lipid kinase to a fraction of its baseline expression, quercetin effectively starves breast cancer cells of the essential downstream phosphoinositide pools required to maintain PIP2-driven focal adhesion assembly, PI3K/Akt/mTOR signaling cascade activation, and cell survival. These findings establish that quercetin operates through a sophisticated dual-action mechanism. It directly blocks the enzyme’s catalytic pocket via competitive binding while simultaneously downregulating its transcription, providing a robust molecular explanation for the observed suppression of migration and growth in aggressive breast cancers.

## 3. Discussion

The findings presented in this study establish *Tamarix aphylla*-derived quercetin as a highly potent, targeted inhibitor of the oncogenic lipid kinase PIP4K2A, expanding the therapeutic boundaries for aggressive breast cancer interventions. Metastatic dissemination remains the primary driver of mortality in triple-negative breast cancer (TNBC), highlighting an urgent need to disrupt non-classical signaling nodes like the PI5P/PIP2 lipid axis [8]. PIP4K2A functions as a critical regulatory hub within this pathway, modulating focal adhesion dynamics to accelerate cellular locomotion [25]. The therapeutic paradigm uncovered here reveals a rare, highly advantageous dual-action mechanism: quercetin simultaneously drives direct, competitive catalytic pocket inhibition and downstream transcriptional gene silencing. This multi-tiered approach effectively blocks compensatory escape pathways typically utilized by tumor cells to resist conventional single-action kinase inhibitors.

At the structural level, the computational data detailed in this study support a profound enzymatic blockade, wherein quercetin achieves an exceptional binding affinity (−10.77 kcal/mol) and outstanding thermodynamic stability ΔG = −42.6 ± 2.1 kcal/mol) within the PIP4K2A ATP-binding pocket, easily outperforming the native co-crystallized ligand. This tight binding is driven by stable, high-occupancy conventional hydrogen bonds formed with hinge-region residues Asn198 and Val199, an architectural arrangement structurally validated by contemporary multi-target screening and docking protocols mapping flavonoid–kinase interactomes, which confirm that the planar chromone scaffold of quercetin stably coordinates within lipid kinase ATP clefts to modulate downstream signaling cascades [26]. Structural hot-spot identification via in silico site-directed mutagenesis provides crucial mechanistic insights into the binding thermodynamics of small-molecule inhibitors. The substitution of hinge residues ASN198 and VAL199 with alanine imposed a notable binding energy penalty ΔG = +2.20 kcal/mol on Quercetin, dropping its binding affinity from −10.77 kcal/mol in the wild-type to −8.57 kcal/mol in the mutant model. Structural analysis demonstrates that this attenuation is primarily driven by the ablation of canonical hydrogen-bonding networks at position 198, which subsequently induced localized pose rearrangement and unfavorable donor-donor electrostatic repulsions with LYS209. Interestingly, core hydrophobic interactions with PHE134, PHE200, and LEU230 remained intact, indicating that while hydrophobic forces maintain basic ligand positioning, specific polar interactions at the hinge region dictate maximum binding potency. Crucially, the co-crystallized ligand maintained strong binding affinity in the double-mutant pocket (−9.90 kcal/mol), confirming that the reduced affinity of Quercetin is ligand-specific rather than a consequence of global active-site distortion. Together, these mutational simulations conclusively define ASN198 and VAL199 as indispensable structural anchors required for high-affinity Quercetin stabilization.

Unrestrained 200 ns molecular dynamics simulations captured a crucial induced-fit structural transition between 15 and 25 ns, where the complex dynamically realigned to lock into an ultra-stable conformation cross-validated by strong anti-correlated movements in Dynamic Cross-Correlation Matrices, thereby driving the system into a deep, single global energy minimum basin (10.8 kcal/mol) that effectively neutralizes the enzyme’s catalytic capacity. In vitro phenotypic assays strongly validate these biophysical models, revealing a clear selectivity for the highly aggressive, mesenchymal triple-negative breast cancer (TNBC) subtype; quercetin exerted potent, dose-dependent cytotoxicity, with MDA-MB-231 cells exhibiting significantly greater sensitivity (IC_50_ = 82.23 µg/mL) than luminal MCF7 cells (IC_50_ = 97.14 µg/mL). This phenotypic preference matches the real-world dynamics of aggressive breast cancers, which frequently undergo extensive metabolic reprogramming; indeed, recent comprehensive profiling emphasizes that the *PIP4K2A* gene is selectively upregulated in advanced malignancies to orchestrate a broad spectrum of oncogenic, metabolic, and invasive processes, creating a unique non-oncogene lipid-kinase addiction in aggressive tumors [27,28]. Scratch wound-healing assays confirmed a dramatic arrest of collective cell migration, reducing absolute wound closure to just ~40%, which single-cell live-tracking clarified is driven by a stark reduction in individual cellular velocity rather than simple growth arrest. By cutting off phosphatidylinositol 4,5-bisphosphate (PI(4,5)P2) generation, quercetin starves the cell of the phosphoinositides necessary to maintain focal adhesion turnover, which aligns with modern mechanical models establishing that localized phosphoinositide pools act as mandatory signaling hubs that integrate integrin trafficking and focal adhesion kinase (FAK) turnover to dictate actin-driven directional cell motility [29,30]. Crucially, the most striking finding in this study is the dual-action advantage of quercetin, characterized by the profound transcriptional silencing of the *PIP4K2A* oncogene, which successfully circumvents a pervasive clinical limitation of targeted small-molecule inhibitors where standard catalytic pocket blockades inadvertently trigger rapid adaptive resistance through target gene mutations and the activation of compensatory signaling feedback loops [31,32]. Instead of inducing a compensatory rebound, quercetin treatment markedly downregulated PIP4K2A expression. RT-qPCR analysis revealed a highly significant reduction, with relative mRNA transcript levels dropping to 0.025-fold compared to the control (*p* < 0.001), a parallel transcriptional and phenotypic reprogramming capacity consistent with recent reports demonstrating quercetin’s unique multi-targeted ability to systematically alter gene expression patterns and induce cell cycle arrest or senescence without triggering target rebound mechanisms [33,34].

Ultimately, by combining immediate catalytic pocket competitive binding with long-term transcriptional silencing, quercetin eliminates the cancer cell’s recovery mechanisms through three core functional pillars established in this study. It enforces an enzymatic blockade that rigidly locks the ATP pocket via an induced-fit transition into a deep thermodynamic basin, drives transcriptional silencing through a near-complete knockdown of *PIP4K2A* mRNA down to 0.025-fold, and induces migratory arrest by reducing individual locomotor velocity to shrink collective wound healing to ~40%. While future genetic knockout and overexpression rescue studies will be valuable to fully confirm absolute mechanistic dependency, our integrated functional assays and MD simulations firmly point to PIP4K2A as a key driver of these effects. Accordingly, this natural flavonoid scaffold represents an exceptional candidate for advanced in vivo profiling and nano-formulation development to optimize its bioavailability against highly motile breast cancer phenotypes.

## 4. Materials and Methods

### 4.1. Plant Material Extraction and Phytochemical Fractionation

Finely pulverized samples of *Tamarix aphylla* (10 g) underwent solid–liquid extraction in a 150 mL methanol/water solvent system (40:60 *v*/*v*) under continuous maceration for 24 h. The crude extract was filtered and concentrated under reduced pressure at 40 °C to a residual volume of 5 mL. To liberate esterified and bound phenolic complexes, alkaline hydrolysis was performed using 5 mL of 2 N NaOH for 30 min followed by neutralization to pH 7.00 2 N HCl. The targeted phenolic fractions were isolated via liquid–liquid extraction with ethyl acetate (3 × 20 mL), and the pooled organic phases were evaporated to dryness. The dry residue was reconstituted in 7 mL of HPLC-grade methanol and filtered through a 0.22 μm syringe membrane before chromatographic profiling [35].

### 4.2. Quantitative High-Performance Liquid Chromatography (HPLC) and FTIR Characterization

Phytochemical fractionation and characterization were executed utilizing a high-resolution SYKAM HPLC system (Sykam GmbH, Eresing, Germany) equipped with a C18-ODS column (25 cm × 4.6 mm). Isocratic elution was strictly maintained at a constant flow rate of 1.0 mL/min using an optimized mobile phase consisting of methanol, distilled water, and formic acid (70:25:5 *v*/*v*/*v*). Eluted components were continuously monitored via a UV-Vis detector set at λ = 360 nm (optimized for the target flavonoid scaffold) and λ = 280 nm. The initial chromatographic profiling of the *Tamarix aphylla* crude extract successfully resolved a distinct multi-component flavonoid profile, identifying the key targeted peaks including Quercetin alongside other co-existing flavonoids (Supplementary Figure S1). To achieve definitive identity confirmation of the targeted isolated fractions and explicitly rule out any co-eluting structural isomers, a rigorous spiking (co-injection) analysis was performed. The isolated fraction was mixed and co-injected alongside a certified commercial Quercetin reference standard (>98% purity). This dual-sample run yielded a single, perfectly symmetrical, sharp chromatographic peak with absolutely no peak-splitting or shoulder anomalies (Supplementary Figure S4c). The precise chromatographic purity of the isolated fraction was quantified by integrating the analytical HPLC peak area, establishing a strict purity profile of 96.4% (Supplementary Figure S4b). Structural validation and structural architecture matching were subsequently confirmed via High-Resolution Fourier-Transform Infrared (FTIR) spectroscopy. The isolated compound exhibited an identical, completely superimposable vibrational fingerprint compared directly to the authenticated commercial standard (Supplementary Figure S4d,e). This includes the precise resolution of diagnostic functional marks, such as the intense phenolic hydroxyl (-OH) stretching modes (3300–3400 cm^−1^), the sharp characteristic carbonyl (C=O) stretching band at ~1660 cm^−1^, and the aromatic skeleton ring vibrations (C=C) at ~1610 cm^−1^.

### 4.3. Receptor Preparation and Active Site Mapping

The high-resolution three-dimensional crystal structure of human PIP4K2A was retrieved from the RCSB Protein Data Bank (PDB ID: 8C8C), co-crystallized with its native ATP-competitive inhibitor: It is a synthetic small-molecule quinazoline-based competitive ATP-site inhibitor (compound ID: 5-chloro-N4-(3,4-dimethylphenyl)-N2-(4-methoxyphenyl)pyrimidine-2,4-diamine) developed to block PIP4K2A catalytic activity. Raw coordinates were prepared and refined using the Protein Preparation Wizard within Schrödinger Maestro. This involved assigning proper bond orders, adding missing hydrogens, optimizing hydrogen-bonding networks at pH 7.0, and remediating missing loops or side chains. Following the deletion of all non-catalytic crystal water molecules, energy minimization of the prepared receptor structure was performed using the OPLS3e force field until the root-mean-square deviation (RMSD) converged within a threshold of 0.30 Å. Finally, the receptor grid box was generated by centering the coordinates on the mass center of the co-crystallized native ligand (Cartesian coordinates: x = −16.88, y = −22.49, z = −6.34).

### 4.4. Molecular Docking and Binding Free Energy (MM/GBSA) Calculations

Molecular docking simulations were executed using AutoDock Vina 1.1 [36], with grid parameters calibrated via AutoDock 4.2 [37] based on the native ligand’s coordinates. Highly exhaustive conformational sampling settings were applied to ensure reliable pose predictions. The final binding modes were ranked according to their binding affinity scores (kcal/mol). The precision of the docking protocol was verified by re-docking the native ligand into the catalytic site, confirming a valid spatial overlap with an RMSD < 2.0 Å [38]. Two-dimensional and three-dimensional ligand-residue interaction networks were mapped using BIOVIA Discovery Studio.

To calculate precise thermodynamic stability, relative binding free energies (ΔG_bind_) were computed using the Molecular Mechanics/Generalized Born Surface Area (MM/GBSA) module in Prime (Schrödinger) [39] under the OPLS3 force field [40], according to the following thermodynamic equation:

$${\Delta G}_{bind}={\Delta E}_{MM}+{\Delta G}_{solv}+{\Delta G}_{SA}$$

where ΔE_MM_ represents the gas-phase molecular mechanics interaction energy, ΔG_Solv_ denotes the generalized Born polar solvation energy, and ΔG_SA_ accounts for the non-polar solvent-accessible surface area energy contribution.

In silico double mutagenesis (N198A/V199A) of the target protein (PDB ID: 8C8C) was performed using BIOVIA Discovery Studio Visualizer; Version 20.1.0.19295, (Dassault Systèmes BIO-VIA, San Diego, CA, USA). Comparative molecular docking of Quercetin and the reference control ligand (CTL) against wild-type (WT) and mutant structures was conducted using AutoDock Vina with identical grid parameters and scoring functions. Intermolecular interactions and 2D/3D binding poses were generated and analyzed using BIOVIA Discovery Studio Visualizer; Version 20.1.0.19295, (Dassault Systèmes BIO-VIA, San Diego, CA, USA).

### 4.5. Molecular Dynamics (MD) Simulations and Trajectory Analysis

Unrestrained molecular dynamics simulations were conducted using Schrodinger Desmond 2021-1 software. To mimic physiological ionic strength, the system was neutralized by adding 150 mM NaCl counterions. Energy minimization was performed using a 5000-step steepest descent algorithm. The system was then equilibrated for 5 ns under both NVT (constant volume) and NPT (constant pressure) ensembles, applying harmonic restraints to the protein backbone.

Finally, a 200 ns production run was executed at 310 K and 1 bar without restraints, utilizing the V-rescale thermostat [41] and Parrinello–Rahman barostat [42]. Trajectory analyses—including RMSD, root-mean-square fluctuation (RMSF), radius of gyration (R_g), and hydrogen-bonding kinetics—were extracted using Simulation Interactions Diagram module of Schrodinger Desmond 2021-1 software.

Principal Component Analysis (PCA) was applied to the backbone covariance matrix to capture essential motions, and the Free Energy Landscape (FEL) was mapped as a function of PC1, PC2, and RMSD. End-state binding energies were verified across the final stable 190–200 ns window [43].

### 4.6. Cell Lines, Culture Maintenance, and In Vitro Cytotoxicity (MTT) Assay

Human breast cancer cell lines (MDA-MB-231 and MCF7) and normal cell line (MCF-10A) were sourced from the American Type Culture Collection (ATCC, Manassas, VA, USA) and verified for experiment safety. Cells were cultured in Dulbecco’s Modified Eagle’s Medium (DMEM) supplemented with 10% fetal bovine serum (FBS, Gibco, Thermo Fisher Scientific, Waltham, MA, USA) and 1% penicillin/streptomycin. The cultures were maintained in a humidified incubator at 37 °C under a 5% CO_2_ atmosphere and routinely screened for mycoplasma contamination using the EZPCR Mycoplasma Test Kit (Geneflow, Lichfield, Staffordshire, UK).

Cell viability was measured using the colorimetric 3-(4,5-dimethylthiazol-2-yl)-2,5-diphenyltetrazolium bromide (MTT) assay [44]. Cells were seeded in 96-well plates and exposed to varying concentrations of quercetin (20–120 µg/mL) for 24 h. Following incubation, 50 µL of serum-free medium and 50 µL of MTT stock solution (5 mg/mL in PBS) were added per well.

After 3 h of incubation at 37 °C the resulting purple formazan crystals were dissolved in 150 µL of DMSO. Absorbance was recorded at lambda = 570 nm using a microplate reader. The IC_50_ values were calculated using non-linear sigmoidal dose–response curves in GraphPad Prism, version 10.0.0.

### 4.7. In Vitro Scratch Wound Healing and Live-Cell Tracking Assays

For collective migration analysis, breast cancer cells were cultivated to >90% confluency in 96-well plates. A standardized wound gap was introduced into the monolayer using a sterile 200 µL pipette tip [45]. Wells were treated with baseline control vehicle (0.1% DMSO) or quercetin at concentrations of 20, 40, 82, and 164 µg/mL. Wound closure kinetics were captured at regular intervals (0, 8, 16, 24, 32, 40 and 48 h) using a Lenovo Q30 digital imaging system mounted on an inverted microscope. Migratory areas were quantified using ImageJ software (version 1.54f; National Institutes of Health, Bethesda, MD, USA) [46]. To assess single-cell kinematics, cells (1 × 10^5^ cells/ml) were monitored via time-lapse microscopy on a Nikon Eclipse Ti-E; Nikon Corporation, Tokyo, Japan [47]. Micrographs were captured every 15 min over a 72 h window. Individual cell trajectories and absolute migration velocities (µm/h) were traced using the MtrackJ plugin in ImageJ [48].

### 4.8. RNA Extraction and Quantitative Real-Time PCR (RT-qPCR)

Total RNA was harvested MDA-MB-231 from both the treated and untreated MDA-MB-231 using the RNeasy Mini Kit (QIAGEN, Hilden, Germany) following the manufacturer’s protocol, and its concentration and purity were verified via a Qubit Fluorometer [42]. First-strand cDNA was synthesized from 1 µg/mL of total RNA template using SuperScript II Reverse Transcriptase (Invitrogen, Thermo Fisher Scientific, Waltham, MA, USA) in a Biometra thermal cycler (AnalytikJena GmbH+Co.Kg, Jena-Göschwitz, Germany).

Oligonucleotide primers targeting human *PIP4K2A* were optimized using Primer3 software version 4.1.0 [49] to operate at a high-stringency melting temperature of 60 °C. The designated primer sets are detailed in Table 2.

Standard curve validation via a 10-fold serial dilution yielded the linear regression profile:

y = −3.17x + 26.247

with a strong coefficient of determination R^2^= 0.8797 The PCR amplification efficiency (E) was determined based on the slope of the regression line using the standard thermodynamic formula [50]:

E = (10^−1/slope^ − 1) × 100

E = (10^0.315^ − 1) × 100

E = (2.065 − 1) × 100

This efficiency value falls well within the strict criteria required for accurate target quantification [51,52]. Real-time quantitative amplification was conducted on a StepOnePlus Real-Time PCR System (Applied Biosystems, Thermo Fisher Scientific, Waltham, MA, USA) using 2x qPCRBIO SyGreen Mix.

The thermal profile consisted of an initial denaturation at 95 °C for 2 min, followed by 40 cycles of 95 °C for 5 s and 60 °C for 25 s, concluding with melting curve analysis (60 °C to 95 °C) to verify amplicon specificity. Relative expression metrics were calculated using the comparative 2^−ΔΔCT^ method [53], normalizing data against *GAPDH* and *18S rRNA* housekeeping controls.

### 4.9. Statistical Evaluation

All quantitative data are expressed as the mean pm standard deviation (SD) derived from at least three independent biological replicates. Statistical comparisons between cohorts were evaluated via one-way Analysis of Variance (ANOVA) followed by post hoc multiple comparison testing using GraphPad Prism. Thresholds for statistical significance were established at * *p* < 0.05, ** *p* < 0.01, and **** *p* < 0.0001.

## 5. Conclusions

This study demonstrates that quercetin, isolated from *Tamarix aphylla*, functions as a potent dual-action inhibitor of the oncogenic PIP4K2A signaling axis in breast cancer cell models. Comprehensive in silico molecular docking and extended 200 ns molecular dynamics simulations showed that quercetin stably engages the ATP-binding cavity and drives the system into a single deep global energy basin through an induced-fit structural transition. These computational findings were validated by in vitro phenotypic assays, where quercetin demonstrated dose-dependent cytotoxicity and significantly suppressed individual cell velocity and collective wound healing, showing high selectivity for the aggressive TNBC line MDA-MB-231. Crucially, RT-qPCR analysis confirmed that quercetin induces near-complete transcriptional silencing of PIP4K2A mRNA expression, down to 0.025-fold. By simultaneously blocking the active site and silencing gene transcription, quercetin effectively disrupts the PI5P/PIP2 lipid signaling cascade essential for tumor progression and metastasis. These insights highlight the potential of this natural flavonoid as a therapeutic scaffold, warranting further in vivo exploration for the management of aggressive, highly motile breast cancer phenotypes.

## Figures and Tables

**Table 1:** Binding scores and RMSD values of Quercetin and CTL against wild-type and mutant PIP4K2A.

Protein Model	Ligand	Binding Affinity (kcal/mol)	RMSD (Å)
8C8C (Wild-Type)	Quercetin	−10.77	1.41
8C8C (Wild-Type)	Co-crystallized ligand	−9.02	1.17
Mutant (N198A/V199A)	Quercetin	−8.57	0.61
Mutant (N198A/V199A)	Co-crystallized ligand	−9.90	1.31

**Table 2:** Optimized Primer Sequences and Physicochemical Parameters for PIP4K2A Real-Time Amplification.

Target Gene	Primer ID	Sequence (5′ → 3′)	T_m_ (°C)	Product Size (bp)
*PIP4K2A*	Pair 1 Forward (F1)	CTGCGGGAGAGGTTTGGAAT	60	159
	Pair 1 Reverse (R1)	GGCCACGTCTTCACTGGTAA	60	
*PIP4K2A*	Pair 2 Forward (F2)	TTACCAGTGAAGACGTGGCC	60	110
	Pair 2 Reverse (R2)	CCGGTACATGCCCAAGAACT	60	

## Data Availability

The original contributions presented in this study are included in the article/Supplementary Materials. Further inquiries can be directed to the corresponding author.

## Supplementary Materials

The following supporting information can be downloaded at: https://www.mdpi.com/article/10.3390/ijms27157063/s1.
