# Supplementary material for: In Vitro Anti-Breast Cancer Effects of Tamarix aphylla-Derived Quercetin and In Silico Insights into Its Targeting of PIP4K2A
Source: Int J Mol Sci. 2026 Aug 6;27(15):7063. doi: 10.3390/ijms27157063 (PMC13466766; doi:10.3390/ijms27157063)
Supplement: Supplementary file 1 [file ijms-27-07063-s001.zip › ijms-4428582-supplementary.pdf]

Supplementary Materials

Chromatography Laboratory
HPLC

Sample Info:

Sample ID	: sample	Amount	0
Sample	: sample	ISTD Amount	0
Inj. Volume [mL]	: 0.1	Dilution	1

Autostop	: 20.00 min	External Start	: Start - Restart, Down
Detector 1	: Detector 3	Range 1	: Bipolar, 2000 mAU, 10 Sample. per Sec.
Subtraction Chromatogram	: (None)	Matching	: No Change

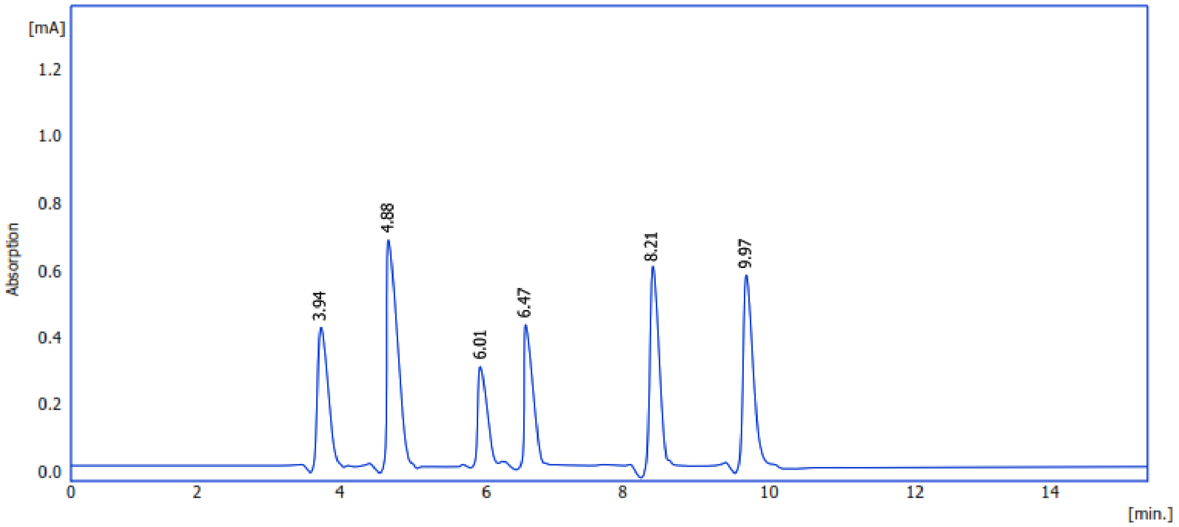

Result chromatography Table (Uncal - F:\ sample)

No	Reten. Time [min]	Area [mAU.s]	Height [mAU]	Area [%]	Height [%]	W 05 [min]	Compound Name
1	3.94	3201.65	400.25	16.00	16.00	0.10	
2	4.88	7412.65	680.65	21.00	21.00	0.20	
3	6.01	2011.45	298.57	13.00	13.00	0.05	
4	6.47	2620.22	377.45	14.00	14.00	0.06	
5	8.21	4123.65	600.12	18.00	18.00	0.07	
6	9.97	4122.69	600.25	18.00	18.00	0.07	
	Total	24125.98	3524.98	100.00	100.00		

Figure S1. HPLC Profile of Crude Extract. High-Performance Liquid Chromatography (HPLC) master chromatogram of the *Tamarix aphylla* crude extract, revealing a multi-component flavonoid profile. Key peaks correspond to Isorhamnetin (~3.94 min), Rutin (~4.88 min), Quercetin (~8.21 min), and Kaempferol (~9.97 min).

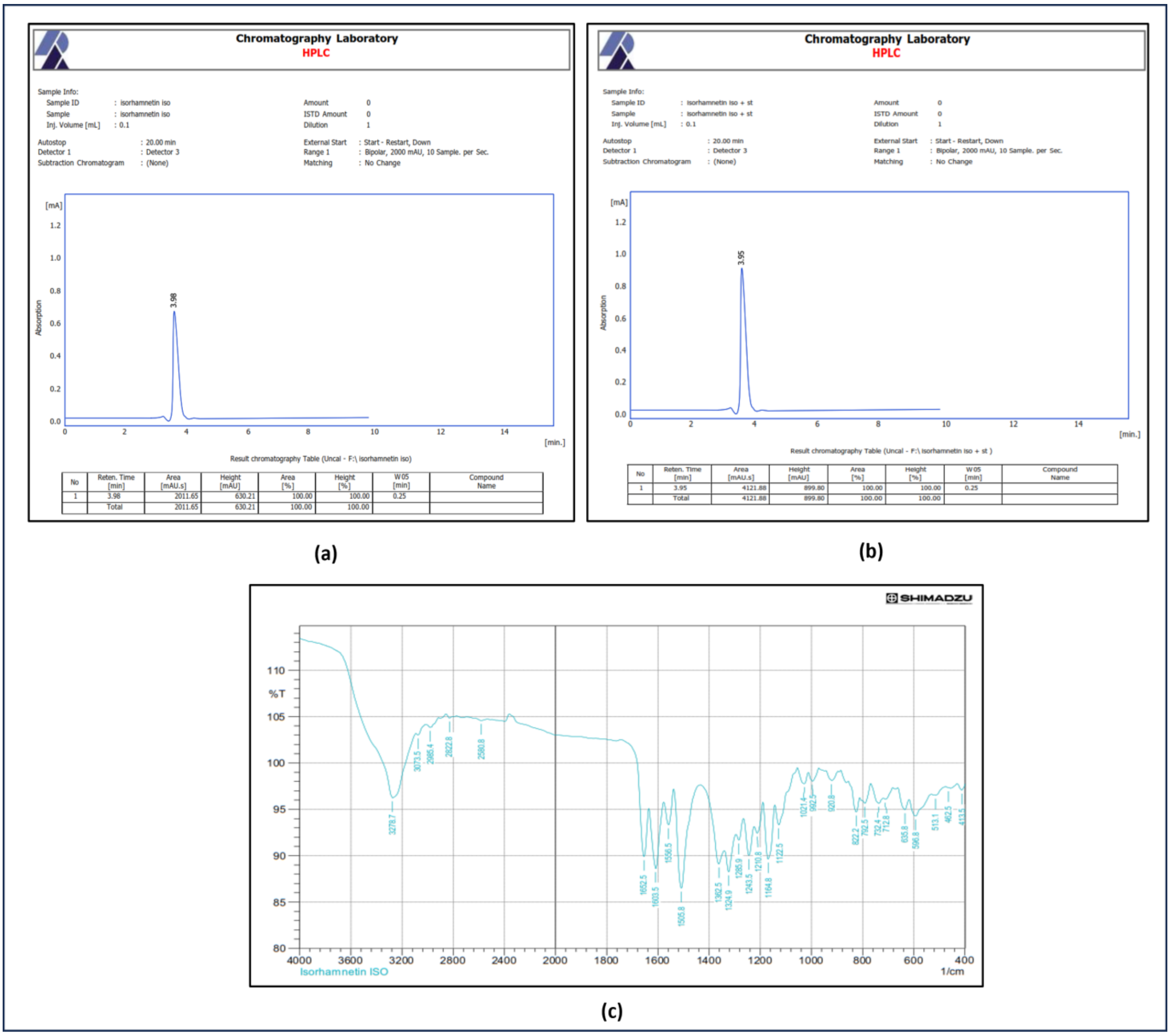

Figure S2. Analytical Characterization of Isorhamnetin. Chromatographic and spectral confirmation of Isorhamnetin. (**a**) HPLC chromatogram of the isolated Isorhamnetin fraction. (**b**) Co-injection HPLC chromatogram of the isolated fraction with a commercial reference standard, confirming peak identity. (**c**) FTIR spectrum of the isolated Isorhamnetin detailing characteristic functional groups.

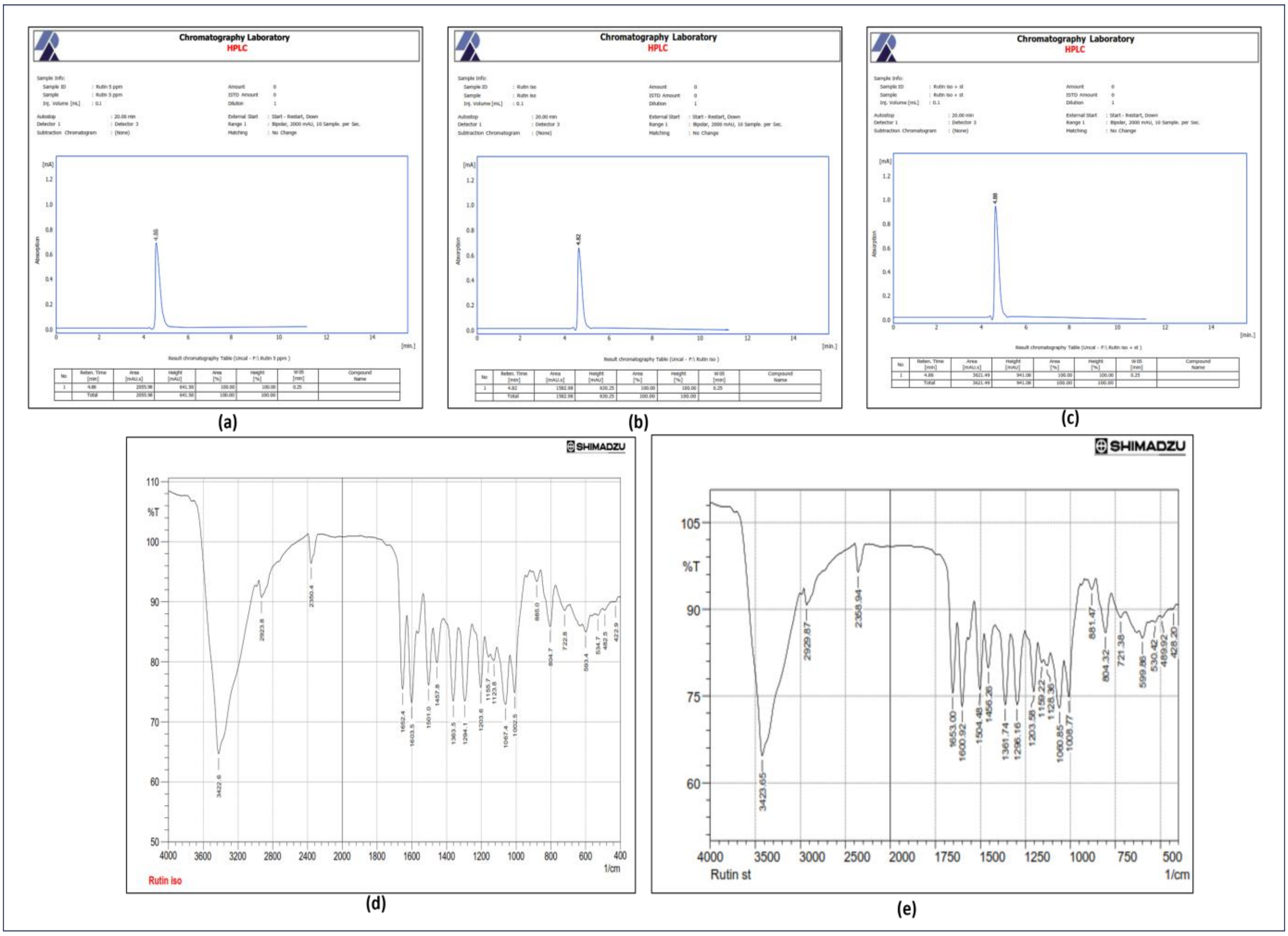

Figure S3. Analytical Characterization of Rutin. Chromatographic and spectral confirmation of Rutin. (**a**) HPLC chromatogram of the highly pure Rutin reference standard (5 ppm). (**b**) HPLC chromatogram of the isolated Rutin fraction. (**c**) Co-injection HPLC chromatogram confirming peak identity. (**d**) FTIR spectrum of the isolated Rutin fraction. (**e**) FTIR spectrum of the commercial Rutin reference standard for structural comparison.

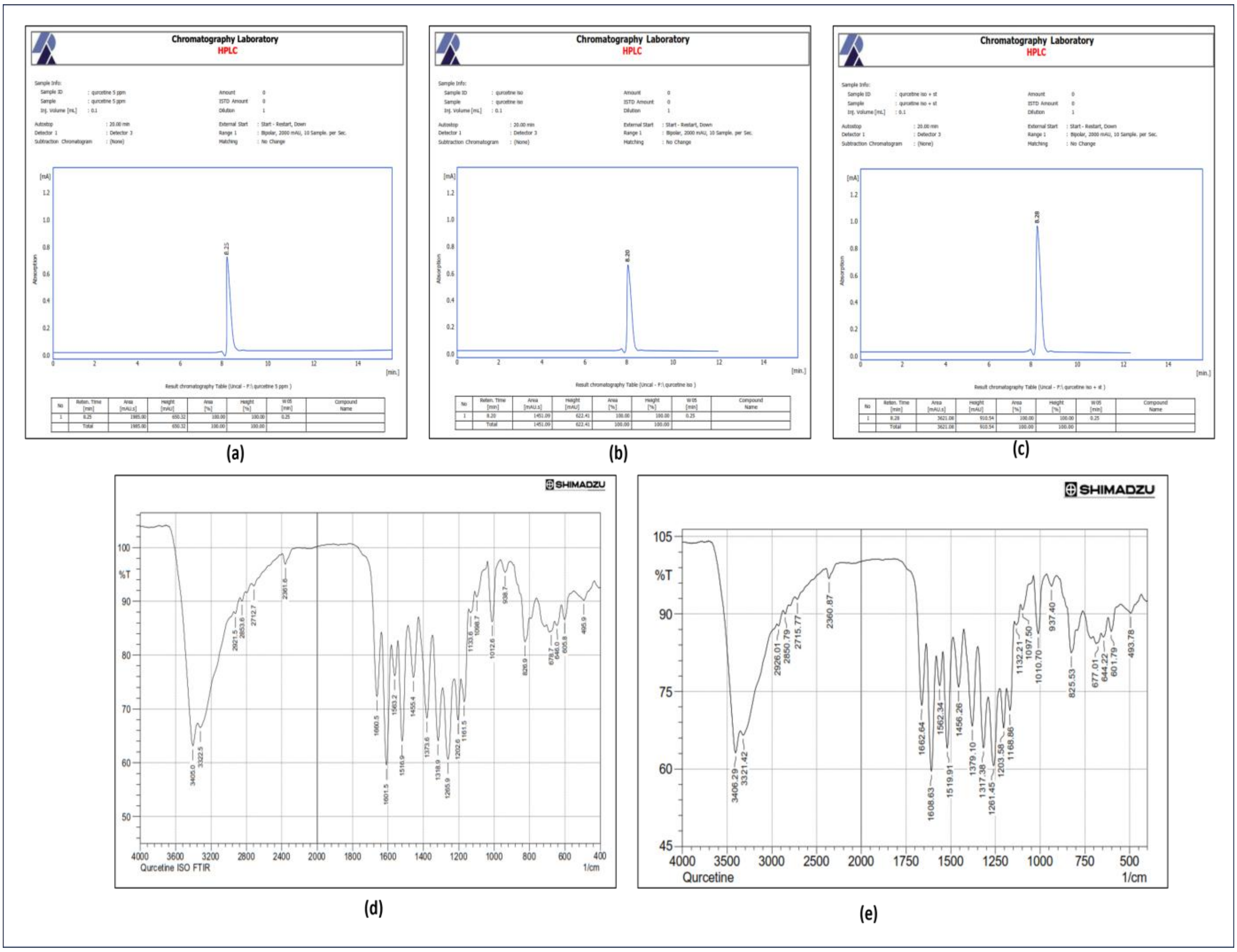

Figure S4. Analytical Characterization of Quercetin. Chromatographic and spectral confirmation of the primary lead compound, Quercetin. (**a**) HPLC chromatogram of the highly pure Quercetin reference standard (5 ppm). (**b**) HPLC chromatogram of the isolated Quercetin fraction. (**c**) Co-injection HPLC chromatogram confirming peak identity. (**d**) FTIR spectrum of the isolated Quercetin fraction. (**e**) FTIR spectrum of the commercial Quercetin reference standard for structural comparison.

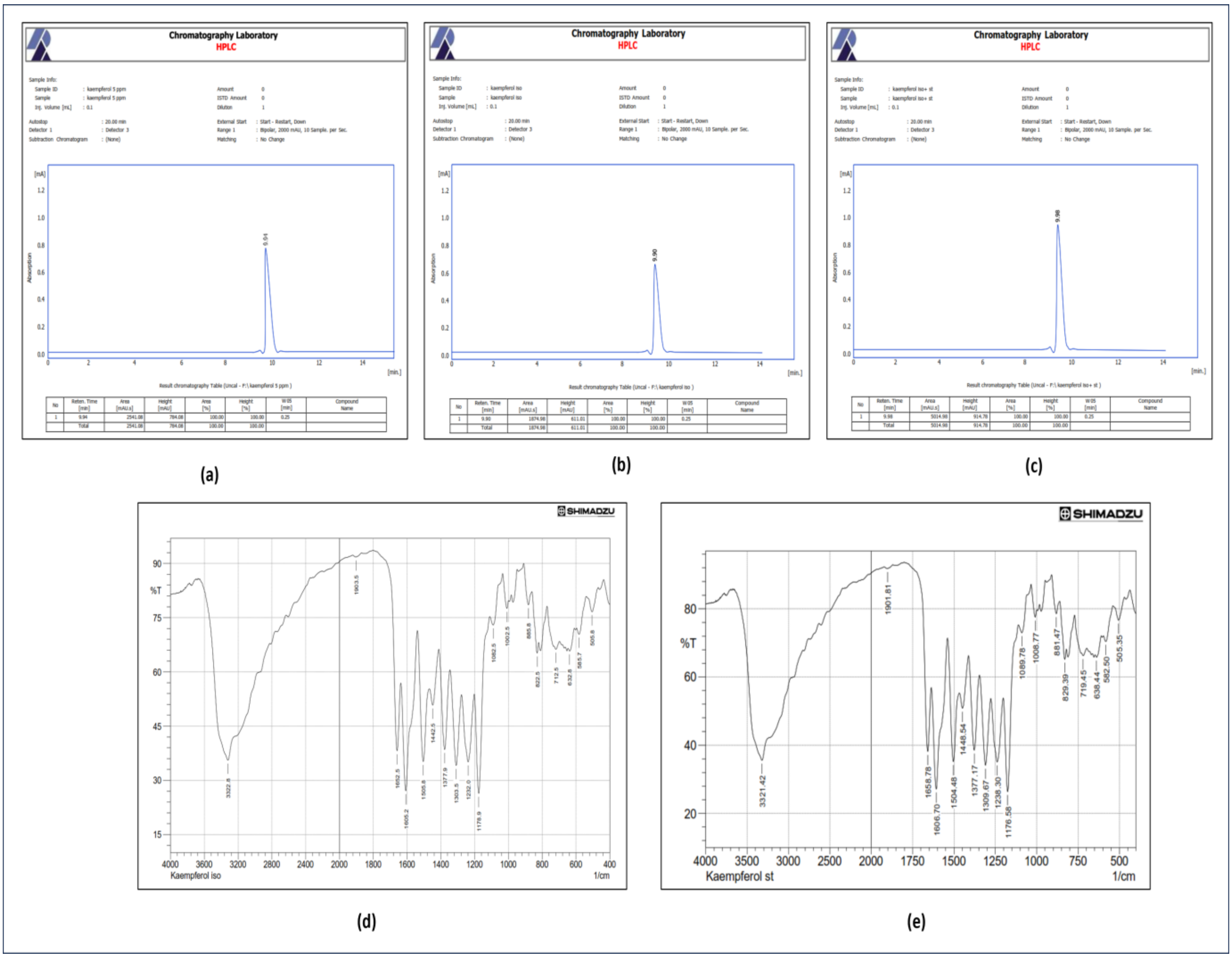

Figure S5. Analytical Characterization of Kaempferol. Chromatographic and spectral confirmation of Kaempferol. (**a**) HPLC chromatogram of the highly pure Kaempferol reference standard (5 ppm). (**b**) HPLC chromatogram of the isolated Kaempferol fraction. (**c**) Co-injection HPLC chromatogram confirming peak identity. (**d**) FTIR spectrum of the isolated Kaempferol fraction. (**e**) FTIR spectrum of the commercial Kaempferol reference standard for structural comparison.

A

B

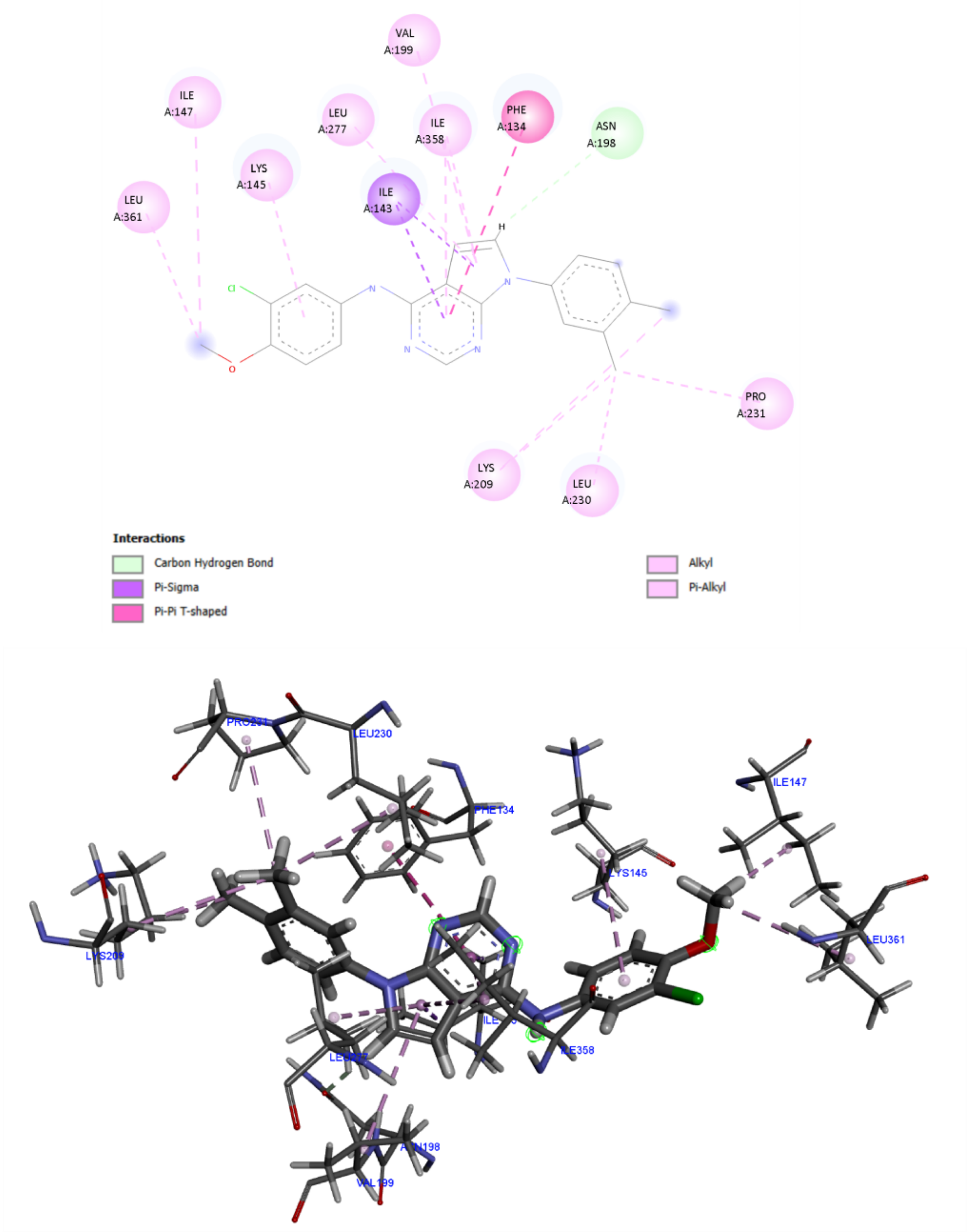

Figure S6. Validation of docking protocol via re-docking of the co-crystallized control ligand into the PIP4K2A binding pocket. (**A**) 2D interaction map illustrating the reproduced binding interactions, including a pi–sigma interaction with ILE143 (purple), a π–π T-shaped interaction with PHE134 (dark pink), a carbon–hydrogen bond with ASN198 (light green), and alkyl π–alkyl hydrophobic contacts with LYS145, ILE147, VAL199, LYS209, LEU230, PRO231, LEU277, ILE358, and LEU361 (light pink). (**B**) 3D binding pose displaying the spatial conformation and non-covalent contacts of the re-docked control ligand within the active site, confirming the accuracy, reliability, and reproducibility of the molecular docking parameters.

A

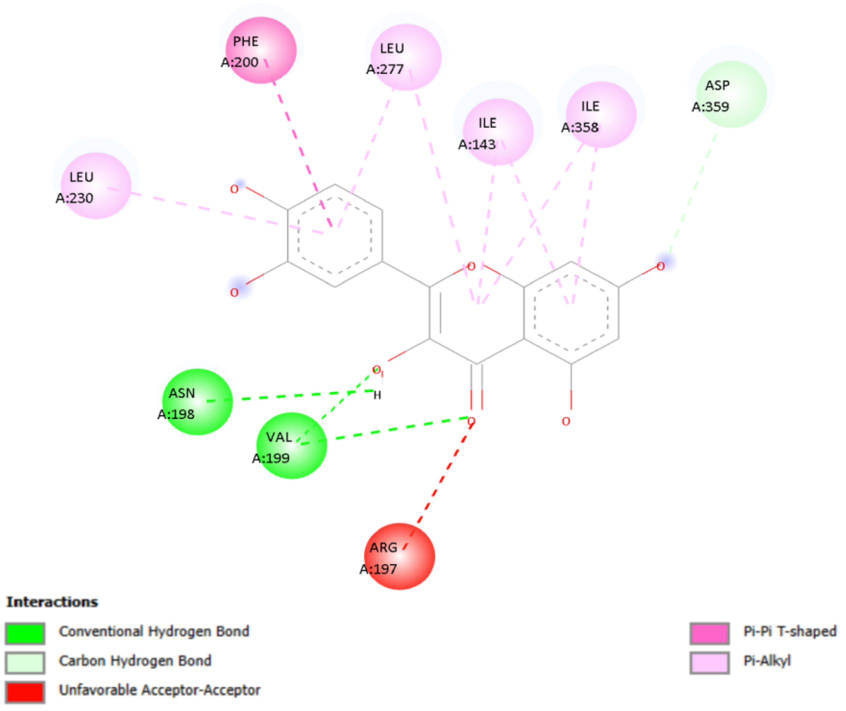

B

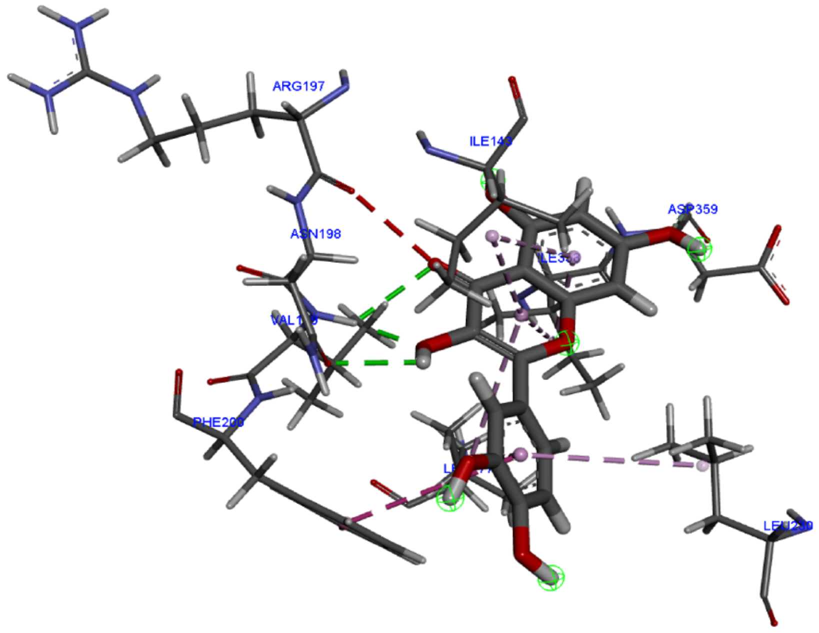

Figure S7. Molecular docking interactions of Quercetin within the active site of PIP4K2A. (**A**) The 2D interaction diagram detailing key non-covalent contacts, including conventional hydrogen bonds with ASN198 and VAL199 (bright green), a carbon–hydrogen bond with ASP359 (light green), a π–π T-shaped interaction with PHE200 (dark pink), π–alkyl hydrophobic contacts with ILE143, LEU230, LEU277, and ILE358 (light pink), and an unfavorable acceptor–acceptor contact with ARG197 (red). (**B**) The corresponding 3D binding pose and spatial orientation of Quercetin within the catalytic pocket.

A

B

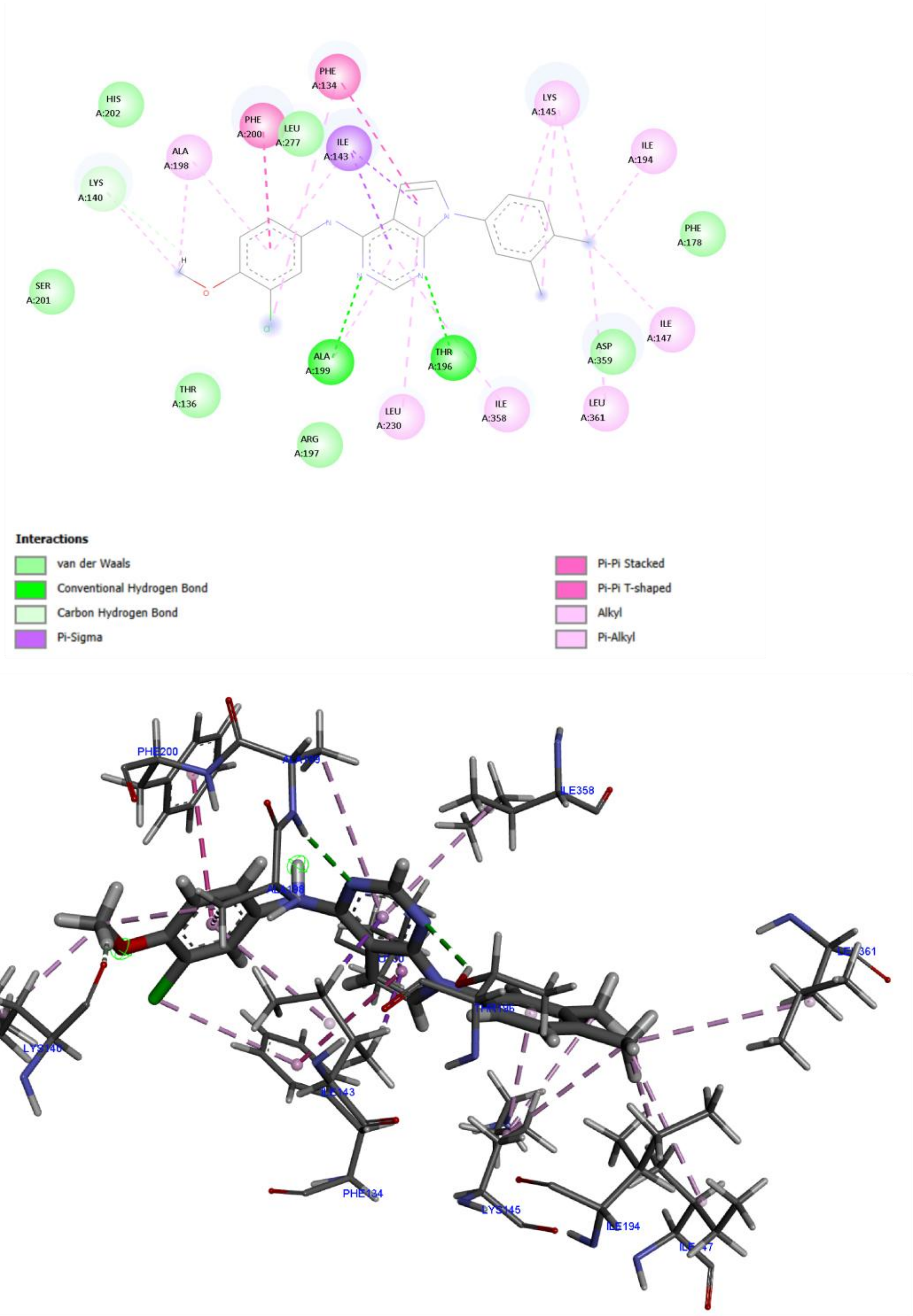

Figure S8. Molecular docking interactions of the control ligand within the double-mutant (N198A/V199A) PIP4K2A active site. (**A**) The 2D interaction diagram detailing key non-covalent contacts, including conventional hydrogen bonds with ALA199 and THR196 (bright green), a carbon–hydrogen bond with LYS140 (light green), a π–sigma interaction with ILE143 (purple), π–π stacked/T-shaped interactions with PHE134 and PHE200 (dark pink), alkyl/π–alkyl contacts with ALA198, LYS145, ILE147, ILE194, LEU230, ILE358, and LEU361 (light pink), and supporting van der Waals interactions (pale green). (**B**) The corresponding 3D binding conformation and spatial orientation of co-crystallized control ligand within the mutated pocket, confirming preserved ligand stability.

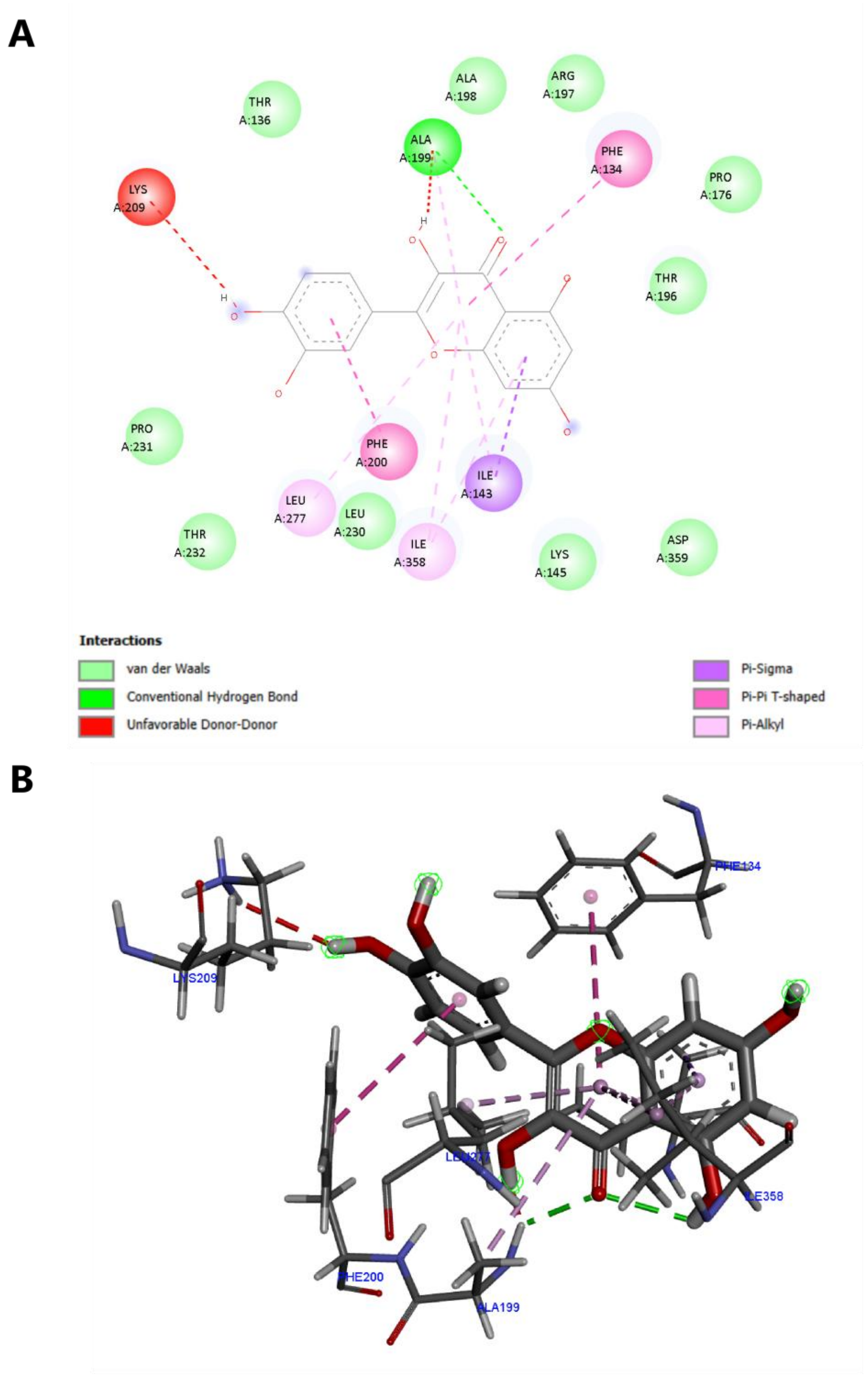

Figure S9. Molecular docking interactions of Quercetin within the double-mutant (N198A/V199A) PIP4K2A active site. (**A**) The 2D interaction diagram detailing key contacts, including a residual conventional hydrogen bond with ALA199 (bright green), unfavorable donor–donor contacts with LYS209 and ALA199 (red), a π–sigma interaction with ILE143 (purple), π–π T-shaped interactions with PHE134 and PHE200 (dark pink), π–alkyl hydrophobic contacts with LEU230, LEU277, and ILE358 (light pink), and surrounding van der Waals interactions (pale green). (**B**) The corresponding 3D binding pose, displaying the spatial rearrangement of Quercetin and steric clashes resulting from the double alanine mutation.
